# The new composition of circulating microvesicles: optimized protocols and reassessment of their characteristics and physiological functions

**DOI:** 10.1093/lifemedi/lnaf017

**Published:** 2025-06-11

**Authors:** Chen Zhang, Jiajia Hu, Yifan Shi, Yang Feng, Zeyang Li, Zi Dong, Yiding Tang, Guang Ning, Zhengting Wang, Guorui Huang

**Affiliations:** Department of Endocrine and Metabolic Diseases, Shanghai Institute of Endocrine and Metabolic Diseases, Ruijin Hospital, Shanghai Jiao Tong University School of Medicine, Shanghai 200025, China; Shanghai National Clinical Research Center for Metabolic Diseases, Key Laboratory for Endocrine and Metabolic Diseases of the National Health Commission of the PR China, Shanghai Key Laboratory for Endocrine Tumor, Ruijin Hospital, Shanghai Jiao Tong University School of Medicine, Shanghai 200025, China; National Research Center for Translational Medicine, State Key Laboratory of Medical Genomics, Ruijin Hospital, Shanghai Jiao Tong University School of Medicine, Shanghai 200025, China; Department of Gastroenterology, Ruijin Hospital, Shanghai Jiao Tong University School of Medicine, Shanghai 200025, China; Department of Nuclear Medicine, Ruijin Hospital, Shanghai Jiao Tong University School of Medicine, Shanghai 200025, China; Department of Endocrine and Metabolic Diseases, Shanghai Institute of Endocrine and Metabolic Diseases, Ruijin Hospital, Shanghai Jiao Tong University School of Medicine, Shanghai 200025, China; Shanghai National Clinical Research Center for Metabolic Diseases, Key Laboratory for Endocrine and Metabolic Diseases of the National Health Commission of the PR China, Shanghai Key Laboratory for Endocrine Tumor, Ruijin Hospital, Shanghai Jiao Tong University School of Medicine, Shanghai 200025, China; National Research Center for Translational Medicine, State Key Laboratory of Medical Genomics, Ruijin Hospital, Shanghai Jiao Tong University School of Medicine, Shanghai 200025, China; Department of Endocrine and Metabolic Diseases, Shanghai Institute of Endocrine and Metabolic Diseases, Ruijin Hospital, Shanghai Jiao Tong University School of Medicine, Shanghai 200025, China; Shanghai National Clinical Research Center for Metabolic Diseases, Key Laboratory for Endocrine and Metabolic Diseases of the National Health Commission of the PR China, Shanghai Key Laboratory for Endocrine Tumor, Ruijin Hospital, Shanghai Jiao Tong University School of Medicine, Shanghai 200025, China; National Research Center for Translational Medicine, State Key Laboratory of Medical Genomics, Ruijin Hospital, Shanghai Jiao Tong University School of Medicine, Shanghai 200025, China; Department of Endocrine and Metabolic Diseases, Shanghai Institute of Endocrine and Metabolic Diseases, Ruijin Hospital, Shanghai Jiao Tong University School of Medicine, Shanghai 200025, China; Shanghai National Clinical Research Center for Metabolic Diseases, Key Laboratory for Endocrine and Metabolic Diseases of the National Health Commission of the PR China, Shanghai Key Laboratory for Endocrine Tumor, Ruijin Hospital, Shanghai Jiao Tong University School of Medicine, Shanghai 200025, China; National Research Center for Translational Medicine, State Key Laboratory of Medical Genomics, Ruijin Hospital, Shanghai Jiao Tong University School of Medicine, Shanghai 200025, China; Department of Endocrine and Metabolic Diseases, Shanghai Institute of Endocrine and Metabolic Diseases, Ruijin Hospital, Shanghai Jiao Tong University School of Medicine, Shanghai 200025, China; Shanghai National Clinical Research Center for Metabolic Diseases, Key Laboratory for Endocrine and Metabolic Diseases of the National Health Commission of the PR China, Shanghai Key Laboratory for Endocrine Tumor, Ruijin Hospital, Shanghai Jiao Tong University School of Medicine, Shanghai 200025, China; National Research Center for Translational Medicine, State Key Laboratory of Medical Genomics, Ruijin Hospital, Shanghai Jiao Tong University School of Medicine, Shanghai 200025, China; Department of Endocrine and Metabolic Diseases, Shanghai Institute of Endocrine and Metabolic Diseases, Ruijin Hospital, Shanghai Jiao Tong University School of Medicine, Shanghai 200025, China; Shanghai National Clinical Research Center for Metabolic Diseases, Key Laboratory for Endocrine and Metabolic Diseases of the National Health Commission of the PR China, Shanghai Key Laboratory for Endocrine Tumor, Ruijin Hospital, Shanghai Jiao Tong University School of Medicine, Shanghai 200025, China; National Research Center for Translational Medicine, State Key Laboratory of Medical Genomics, Ruijin Hospital, Shanghai Jiao Tong University School of Medicine, Shanghai 200025, China; Department of Endocrine and Metabolic Diseases, Shanghai Institute of Endocrine and Metabolic Diseases, Ruijin Hospital, Shanghai Jiao Tong University School of Medicine, Shanghai 200025, China; Shanghai National Clinical Research Center for Metabolic Diseases, Key Laboratory for Endocrine and Metabolic Diseases of the National Health Commission of the PR China, Shanghai Key Laboratory for Endocrine Tumor, Ruijin Hospital, Shanghai Jiao Tong University School of Medicine, Shanghai 200025, China; Department of Gastroenterology, Ruijin Hospital, Shanghai Jiao Tong University School of Medicine, Shanghai 200025, China; Department of Endocrine and Metabolic Diseases, Shanghai Institute of Endocrine and Metabolic Diseases, Ruijin Hospital, Shanghai Jiao Tong University School of Medicine, Shanghai 200025, China; Shanghai National Clinical Research Center for Metabolic Diseases, Key Laboratory for Endocrine and Metabolic Diseases of the National Health Commission of the PR China, Shanghai Key Laboratory for Endocrine Tumor, Ruijin Hospital, Shanghai Jiao Tong University School of Medicine, Shanghai 200025, China; National Research Center for Translational Medicine, State Key Laboratory of Medical Genomics, Ruijin Hospital, Shanghai Jiao Tong University School of Medicine, Shanghai 200025, China

**Keywords:** circulating microvesicles, optimized protocols, extracellular vesicles

## Abstract

Microvesicles (MVs) have convenient clinical applications and play functional roles in cellular signal transduction. Although the clinical importance of MVs is being increasingly recognized, the current diversity of isolated protocols results in a heterogeneous population of their unknown origins, even expands to uncertain functions. Here, we systematically investigated the composition of MVs at different centrifugal speed intervals and discovered that centrifugation at 3000 *g* is critical in determining the composition of MVs. We observed that platelet-derived particles accounted for more than 80% of MVs under 3000 *g*, while only about 20% of MVs were obtained over 3000 *g*. The discovery that more than 80% of platelet-derived MVs sheds new light on their function, including procoagulation activity and clinical diagnosis, etc. Our work not only optimizes the method for MVs isolation but also clarifies the physiological functions and characteristics that should be attributed to platelets rather than MVs. Consequently, these findings will derive new conceptualizations regarding MVs’ composition and function.

## Introduction

Extracellular vesicles (EVs) can be classified into exosomes, microvesicles (MVs) (or microparticles), and apoptotic bodies based on their sizes and synthesizes [1, 2]. MVs are heterogeneous plasma membrane vesicles with diameters ranging from 50 nm to 1000 nm, generated by various cells such as platelets, endothelial cells, leukocytes, and erythrocytes, and so on, through the budding of the outer cell membrane during cell activation or apoptosis [3–5]. As an additional mechanism for intercellular communication, MVs enable cells to exchange proteins, lipids, and genetic material [3]. Accordingly, there is a growing evidence that MVs play roles in a range of processes [6, 7], including procoagulant and fibrinolytic activities [8], vascular remodeling or neoangiogenesis [9], promotion of cellular interactions and signal transmission [10], and provision of malignant cell microenvironments [11]. However, the methods used to isolate MVs currently lack standardization, and their results are sometimes inconsistent or conflicting [12–16]. The process of MVs isolation usually involves several steps, including low-speed centrifugation to remove cells, platelets, and apoptotic debris, followed by high-speed centrifugation to precipitate MVs [17]. Common centrifugation parameters used for MVs isolation vary from 1000 to 5000 *g* for 5–20 min in the initial centrifugation step, followed by 13,000–100,000 *g* for 30–60 min to pellet MVs [12, 18]. Therefore, the characteristics of MVs depend on the detection protocols used, as there is no uniform consensus on the definition of MVs [19]. To avoid false quantification of the composition and understand the real function of MVs, optimization and standardization of detection methods are crucial.

Platelets play an active role in the immune response to microbial organisms and foreign substances by triggering platelet–neutrophil interactions [20, 21]. Recent studies have shown that platelet activation also leads to circulating MVs formation in addition to the well-known hemostatic and inflammatory responses, which is becoming an increasing focus in medical research [22–24]. MVs were first described as subcellular material originating from platelets in normal plasma and serum, formerly known as ‘platelet dust’ [24]. It has long been thought that 70%–90% of MVs in plasma are derived from platelets [17, 25, 26]. MVs have been studied mainly for their role in blood coagulation [27], vascular remodeling or angiogenesis [9], which are similar to the functions of platelets. Therefore, according to the current mainstream MVs detection protocols, the vast majority of MVs are likely be derived from platelets, and thus their functions also depend on platelets. This obscures the characteristics and functions of many other cell-derived MVs and interferes with the results and conclusions of MVs studies.

In addition, high levels of MV have been associated with various diseases, and shown their diagnostic and prognostic value [28–31]. However, despite decades of extensive studies, clinical benefits from MVs alterations in patients have not been demonstrated, partly because the vast majority of “platelet-derived MVs” obscure the characteristics and functions of many other cell-derived MVs. To address this issue, we investigated the proportion of MVs derived from different cells at different centrifugal speeds. Our systematic studies found that the proportion of MVs from different cell sources was relatively stable after 3000 *g*, indicating that the MVs obtained after this point may be true MVs. After that, further confirmation by electron microscopy, dynamic light scattering (DLS), immunofluorescence combined with flow cytometry, and other techniques showed that the proportion of MVs derived from each cell type was very stable after 3000 *g*. Finally, we re-evaluated and found that some functions belong to platelets rather than MVs, such as procoagulation activity, while others belong to both platelets and MVs, such as anti-inflammatory effects (but MVs were better). Some functions, such as the promotion of cell migration and tube formation, benefited from other compositions of MVs, while others, such as endothelial cell-derived MVs, were useful for the clinical diagnosis of cardiovascular disease. Thus, our works have established a method for isolating true MVs and provide a basis for future studies on their characteristics and physiological functions.

## Results

### Diverse and confusing MVs isolation methods

Recent studies have shown that MVs contain mitochondrial components as well as intact mitochondria, which have been described as mediators of inflammation [32, 33]. Therefore, we detected the mitochondrial components from different cells and found that the proportion of mitochondrial characteristic MVs from different cells was almost similar (Fig. S1A). However, we also found that the proportion of MVs derived from platelets (CD41a^+^) was dozens of times higher than those from other cell sources (Fig. S1B). This raised our suspicion that the current methods used for isolating MVs may have inherent issues. It is possible that many of the particles identified as “MVs” could actually be small platelets or large platelet fragments, which could interfere with the staining for mitochondrial-positive MVs. These “MVs” may not be generated through physiological processes, as their membrane of particles originates from the plasma membrane of platelets, rather than through the normal budding of various membranes.

Although MVs have been extensively studied and found to have diverse functions, there is a lack of a uniform standard for their isolation methods (Table S1). In particular, the initial centrifugation speed for removing cell fragments is rarely the same, resulting in a large difference in the composition of MVs. However, most of the MVs are platelet-derived, accounting for 70%–90%. The diversity of components naturally leads to the diversity of their functions, and some of them even have contradictory functions (Table S1). For example, they have been reported to promote tumor metastasis [34] but have also been reported to inhibit cell migration and proliferation [35]. While most published studies on MVs have focused on their potential functions rather than their origins, it is still unclear which cell-derived particles are responsible for any given function [3]. Therefore, to clarify these confusions and uncertainties about MVs studies, it is necessary to systematically study the composition of MVs at different centrifugation speeds and redefine their physiological functions.

### CD41a^+^ MVs obtained at different initial centrifugal speeds

**Figure 1.**
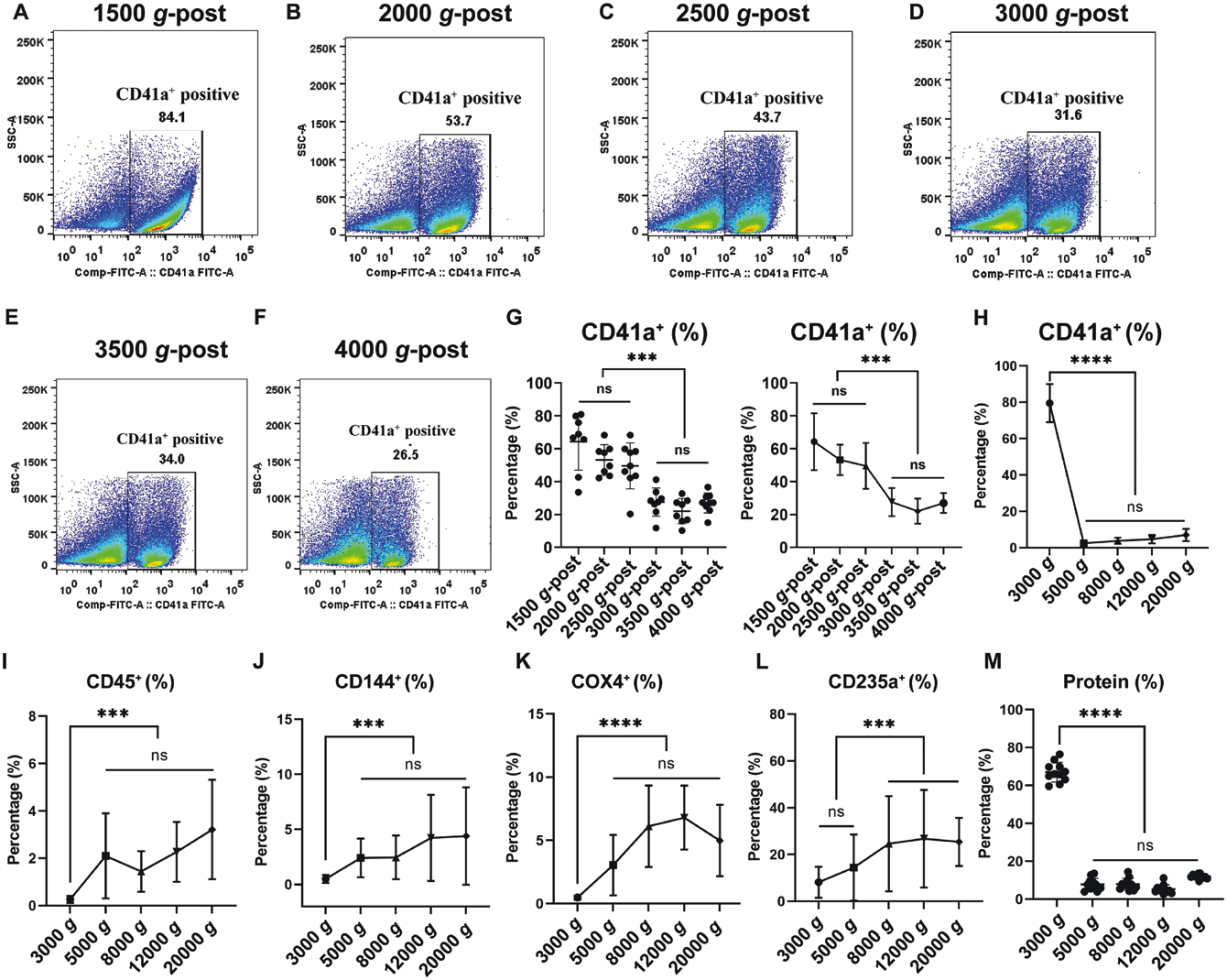
The proportion of CD41a^+^ MVs isolated after different initial speeds, and detection of circulating cell-derived MVs at centrifugal speed intervals. (A–F) Representative images of flow cytometry analysis for CD41a^+^ MVs were isolated at 20,000 *g* after initial concentration speeds of 1500 *g* (A), 2000 *g* (B), 2500 *g* (C), 3000 *g* (D), 3500 *g* (E) and 4000 *g*-post (F), respectively. (G) The percentage of CD41a^+^ were analyzed and its run charts were shown. (H–L) After being centrifugated under different speeds intervals, the percentage of platelet-derived (CD41a^+^) (H), leukocyte-derived (CD45^+^) (I), vascular endothelial cells-derived (CD144^+^) (J), mitochondrial-derived (COX4^+^) (K) and erythrocyte-derived (CD235a^+^) MVs (L), were shown, respectively. (M) The percentage of proteins obtained at five different centrifugation speeds relative to total particles proteins. *n *= 8–10 per group, data are presented as mean ± SD. ***P < 0.001, ****P < 0.0001.

Although the initial centrifugal speed varies in different literatures, most of them were centrifuged at around 1500 *g* for 20 min to remove the cells and cell debris, and then harvested the EV pellets at around 20,000 *g* for 30 min. To investigate the optimal centrifugal speed for isolating the stable MVs, particles were isolated at 20,000 *g* after different initial concentration speeds: 1500 *g*, 2000 *g*, 2500 *g*, 3000 *g*, 3500 *g,* and 4000 *g*-post, respectively (Figs. 1A–F, S2). The data showed that the proportion of CD41a^+^ decreased with higher centrifugal speeds, and it became consistently stable after 3000 *g*-post (Fig. 1G). Similarly, MVs derived from endothelial cells also exhibited a gradual stabilization after 3000 *g* centrifugation (Fig. S5D). This suggests that 3000 *g* may be the critical centrifugal speed for the composition of MVs.

### MVs obtained at different centrifugal speed intervals

Furthermore, we isolated particle samples at different centrifugal speed intervals after an initial centrifugation at 1500 *g*, 3000 *g*, 5000 *g*, 8000 *g*, 12,000 *g*, and 20,000 *g*. And then different cells-derived particles were detected with flow cytometry, the negative and positive controls were shown in Fig. S3. Consistently, over 80% of the particles were CD41a^+^ positive under centrifugation at 3000 *g*. However, only about 20% CD41a^+^ MVs were found at all other centrifugal speed intervals, expressing a stable proportion (Figs. 1H and S4A). This suggests that centrifugation at 3000 *g* for 20 min effectively removes small platelets and platelet fragments, leaving behind the true platelet-derived-MVs. In contrast, compared to the mere 0.26% of CD45^+^ (leukocyte-derived) MVs obtained at 3000 *g*, the percentage of CD45^+^ MVs isolated from other centrifugal speed intervals after 3000 *g* was about 2.2%, approximately 10 times higher, and remained relatively stable (Figs. 1I and S4B). Similarly, endothelial-derived MVs (CD144^+^) obtained at 5000 *g* and higher centrifugation speeds (about 3%) were approximately six times higher than at 3000 *g* (about 0.5%) and were also very stable (Figs. 1J and S4C). Meanwhile, when using another endothelial marker, CD31, we observed the same trend (Fig. S5B).

Furthermore, due to the presence of a large number of non-MVs particles, the proportion of mitochondria-positive MVs harvested at 3000 *g* was very low, whereas it increased to around 3%–7% when centrifuged at higher than 3000 *g*, which is significantly higher than that obtained at 3000*g* (Figs. 1K and S4D). Interestingly, the erythrocyte-derived MVs (CD235a^+^) obtained at 5000 *g* were only slightly higher than those obtained at 3000 *g*, but the difference was not significant. However, obtaining CD235a^+^ MVs was much higher when centrifuged at speeds over 5000 *g* (Figs. 1L and S4E). In contrast, MVs identified by another erythrocyte marker, CD71, showed the opposite trend, with higher yields at 3000 *g* and a gradual decrease at higher centrifugation speeds (Fig. S5A). This discrepancy may be due to that CD71 is mainly expressed on immature erythrocytes and reticulocytes, making CD71^+^ MVs more prone to release at lower centrifugation speeds, whereas CD235a is a marker of mature erythrocytes, requiring higher centrifugation forces to efficiently isolate CD235a^+^ MVs. When considering that the proportion of MVs from different cell sources, it remained relatively consistent at centrifugal speeds above 3000 *g*, and significantly higher than that obtained at 3000 *g*, except for CD41a^+^ (Figs. 1H–L, S4). Additionally, particle samples harvested under 3000 *g* showed a 10-fold higher protein concentration compared to the other groups, indicating that the majority of MV proteins obtained after 1500 *g* were from the 3000 *g* fraction (Fig. 1M). These findings suggest that when intact cells and platelets were removed at 1500 *g* for 20 min, the directly collected MVs mainly consisted of platelet fragments, small platelets, and some erythrocyte debris. However, after an additional 20 min of centrifugation at 3000 *g*, the debris was largely eliminated, and the resulting plasma particles represented are the stable and genuine cell-derived MVs, even harvested at different speed intervals. It indicated that 3000 *g* is a critical centrifugation speed in determining the composition of MVs.

### Analysis of MVs by combining with immunofluorescence and flow cytometry

To further validate the above results from flow cytometry, we combined immunofluorescence and flow cytometry to investigate the fractions of EV in plasma obtained at different centrifugation speeds. We labeled the MVs with detection antibodies for cellular markers and used secondary antibodies that emitted red fluorescence. Consistent with the results obtained previously, direct observation by confocal microscopy revealed that platelet-derived MVs obtained at 3000 *g* after 1500 *g* accounted for the majority of the MVs (Fig. S6A). This was significantly higher than those from other cell sources, such as leukocytes and erythrocytes (Fig. S6A–C). The same samples were also quantitatively analyzed by flow cytometry again, and the results were similar to those presented in (Figs. 1I–M, S6D).

In addition, Western blotting was used to further confirm the results, and we found that the bands of platelet marker proteins CD41 and CD36 in the 3000 *g* sample were much stronger than those obtained at other centrifugation speeds (Fig. S6E). Importantly, the markers of other cells, such as erythrocytes (glycophorin), leukocytes (CD45), and membrane lipoprotein (Perilipin1), obtained at different speeds did not differ significantly. However, as a cytoskeletal protein marker in the cytosol, the band of Actin in the 3000 *g* sample was much stronger than that in other centrifugation speeds, which was similar to the platelet markers CD41 and CD36 (Fig. S6E). This further suggested that most of the 3000 *g* particles were large cellular fragments or debris, and a small number of erythrocyte fragments, rather than MVs.

### The proportional MVs with centrifugal speeds

**Figure 2.**
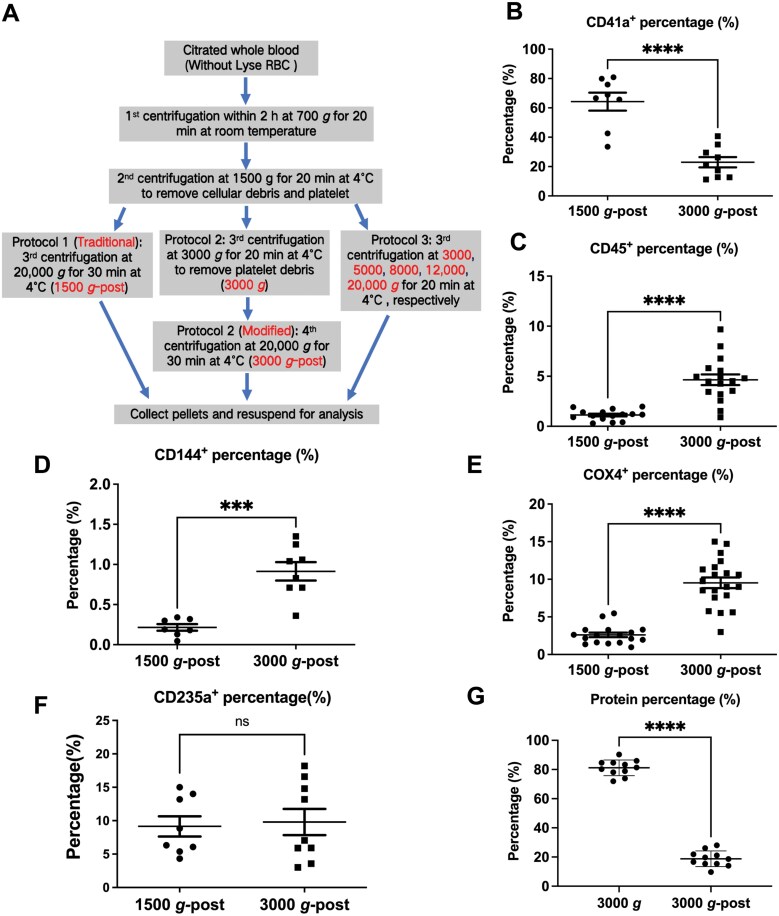
The percent of the MVs compositions and the amount of protein according to 1500 *g*-post and 3000 *g*-post centrifugation. (A) The summarized protocols for the isolation of circulating particles. (B–F) The percentage of platelet-derived (CD41a^+^) (B), leukocyte-derived (CD45^+^) (C), vascular endothelial cells-derived (CD144^+^) (D), mitochondrial-derived (COX4^+^) (E), and erythrocyte-derived MVs (CD235a^+^) (F) were shown, which obtained at 1500 *g*-post and 3000 *g*-post. (G) The percentage of protein after being centrifuged at 3000 *g* and then 20,000 *g* (3000 *g*-post) one after another, the total protein is equal to the protein of 1500 *g*-post. Data are mean ± SD; *n* > 10 per group. ***P < 0.001, ****P < 0.0001.

Although the initial centrifugal speed varies in different literatures, most of them centrifuge at around 1500 *g* for 20 min to remove cells and cell debris, and then harvest the MVs pellet at around 20,000 *g* for 30 min. To investigate how the initial centrifugal speed affects the proportions of MVs, we compared the 1500 *g* (1500 *g*-post) and 3000 *g* (3000 *g*-post) as the initial speed to remove cell debris, respectively. As shown in Fig. 2A, the protocols for the isolation of circulating particles were summarized. As expected, over 70% of the pellets were platelet-derived (CD41a^+^) for 1500 *g*-post as in previous reports (Fig. 2B). However, only about 20% platelet-derived MVs were found when they were harvested at 3000 *g*-post, which is 2–3 times less than 1500 *g*-post. Conversely, all of the other cell-derived MVs like leukocyte (CD45^+^) (Fig. 2C), vascular endothelial cells (CD144^+^) (Fig. 2D), and mitochondrial-derived MVs (COX4^+^) (Fig. 2E) increased over 5-folds in 3000 *g*-post compared with 1500 *g*-post group. Interestingly, no significant increase was found in 3000 *g*-post erythrocyte-derived MVs (CD235a^+^) (Fig. 2F), most likely because there was still some erythrocyte-derived debris present in the 1500 *g*-post particles. Besides, the total protein of MVs obtained at 3000 *g* was detected around 4-fold the amount of 3000 *g*-post (Fig. 2G). It also means that the amount of EV proteins obtained from 3000 *g*-post was only about 20% of that of 1500 *g*-post, and about 80% of the proteins in 1500 *g*-post could be removed at 3000 *g.* It indicates that most of the so-called “MVs” obtained by 1500 *g*-post are mainly derived from small platelets and large platelet debris, and some of them also derive from erythrocyte fragments. These results further demonstrate that the centrifugal speed plays a critical role in MVs composition, and 3000 *g* is the boundary centrifuged speed for MVs isolation.

### Morphological characterization and size of MVs

**Figure 3.**
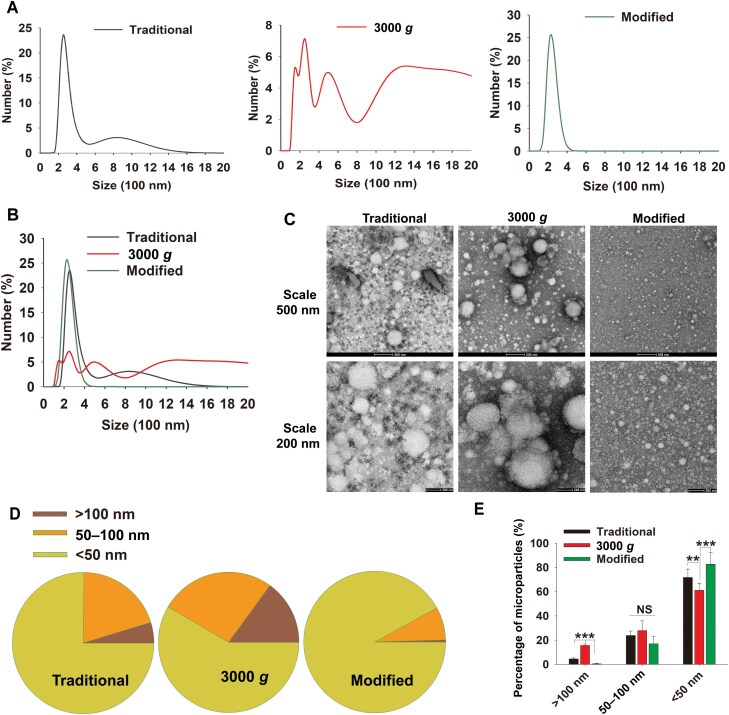
Morphological characteristics and size of MVs. (A) The size distribution of MVs was analyzed by the DLS for samples which obtained with traditional (1500 *g*-post), 3000 *g* and modified (3000 *g*-post) protocols. (B) The three sizes distribution of MVs were merged. (C) Representative images were shown for particles morphological analysis by negative stain TEM. (D) Statistic analyses for the proportion of different sizes of particles. (E) Percentages of MVs were quantified for different size distributions from three different methods. Data are mean ± SD; over 10 images per group were analyzed. **P < 0.01, ***P < 0.001.

Size is a fundamental characteristic of MVs, with a recognized size range of about 50–1000 nm, which is very wide [3, 4]. To further understand the size of MVs obtained by the 3000 *g*-post method, we first used dynamic light scattering (DLS) to detect the size distribution of MVs obtained by 1500 *g*-post (traditional), 3000 *g,* and 3000 *g*-post (modified), respectively. The data showed that the MVs obtained by the traditional method had two obvious peaks (Fig. 3A). In terms of the proportion of number, the main peak was mainly at 150–500 nm (about 85%), while another weaker peak was around 600–1600 nm (about 15%) (Fig. 3A). However, this weaker peak constituted about 80% amount of the total protein (Fig. 1N). Interestingly, the “MVs” obtained at 1500 *g* and then at a relative low speed of 3000 *g* showed multiple peaks, especially the “MVs” over 1000 nm showed a plateau peak of about 5% until over 2000 nm particles (total about 48.9%) (Fig. 3A). Although there was also a peak in the 100–500 nm region of 3000 *g* particles, the total number proportion was only about 40%. There was also a peak at 500–800 nm, which accounts for about 11% (Fig. 3A). What was more interesting was that the MVs obtained after our improved separation method had only one main peak at 100–500 nm, and its number accounted for 99% (Fig. 3A). Additionally, the superposition of the size distribution of particles by the three different methods also showed obvious differences (Fig. 3B). All of these results showed that the size of MVs obtained by the modified protocol had good uniformity and a unique distribution, suggesting that our improved method is excellent for the definition of MVs without causing confusion, generality and uncertainty.

To observe the particles more intuitively, we used transmission electron microscopy (TEM) with negative staining to analyze their morphology and size. Consistent with the DLS results, the MVs obtained by the traditional method had both large and small particles, while the “microparticle” obtained by 3000 *g* had greatly reduced small particles (Fig. 3C). However, the MVs obtained by the modified method showed pretty uniformity and uniqueness (Fig. 3C). Various statistical analyses had shown significant differences from the traditional methods (Fig. 3D–E). Herein, it should be pointed out that the size of the MVs observed by TEM was smaller than that detected by the DLS method. We speculated that this was mainly because the MVs need to be fixed during the preparation of the electron microscope samples, then the MVs were negatively stained and dried with a heat lamp. All of these factors resulted in the shrinkage of plasma particles and a significant reduction in the observed sample size due to the membrane structure of particles. This speculation was confirmed by DLS data, which showed the size of the samples much smaller after being prepared as the above TEM methods (Fig. S7). Thus, the actual size of MVs should be determined by the DLS method without fixation, and the particles were resuspended in solution. In view of this, the size of new MVs should be within the 100–500 nm range, and the vast majority are distributed in a narrower range between 200–350 nm (about 85%).

### Biochemical detection of cell-derived MVs

To further analyze the characteristics of MVs with different methods, we used biochemical methods such as immunofluorescence combined with flow cytometry and Western blotting. As previously mentioned, particles isolated with the traditional method (1500 *g*-post) were similar to those obtained by centrifugation at 3000 *g*, and the percentage of CD41a^+^ particles was over 80%, which was significantly higher than that obtained by our improved method (about 30%) (Fig. S8A and S8D). Besides, particles obtained at 1500 *g*-post and 3000 *g* were more abundant and existed in agglomerate, rather than being smaller and dispersed as in 3000 *g*-post MVs (Fig. S8A–C). Similarly, for leukocyte- and erythrocyte-derived MVs, both traditional and modified methods did not differ significantly, but they all had a significantly lower proportion of positive particles obtained at 3000 *g* (Fig. S8D).

Furthermore, we detected various cell-derived proteins (derived from an equal volume of plasma) by Western blotting. It was observed that the platelet-derived protein CD41 in 3000 *g*-post samples was significantly lower than that in 1500 *g*-post and 3000 *g* samples. Notably, marker proteins such as Perilipin1 (lipid protein), Glycophorin (RBC marker), and CD45 (WBC marker) showed no significant differences between the modified and traditional methods. In contrast, the levels of these proteins were significantly higher in the modified method compared to the 3000 *g* sample (Fig. S8E). More importantly, as Actin is a cytoskeletal protein marker present in the cytoplasm, we found that its expression was significantly higher in the 1500 *g*-post or 3000 *g* group, than in our improved method, further indicating that particles in 1500 *g*-post and 3000 *g* mainly originated from cell fragmental particles rather than plasma membrane-dominant vesicles (Fig. S8E). These data were consistent with previous results and demonstrated that the majority of proteins isolated by traditional methods were platelet-derived particles and platelet fragments rather than plasma membrane vesicle MVs.

### Procoagulant function belongs to platelet granules rather than MVs

**Figure 4.**
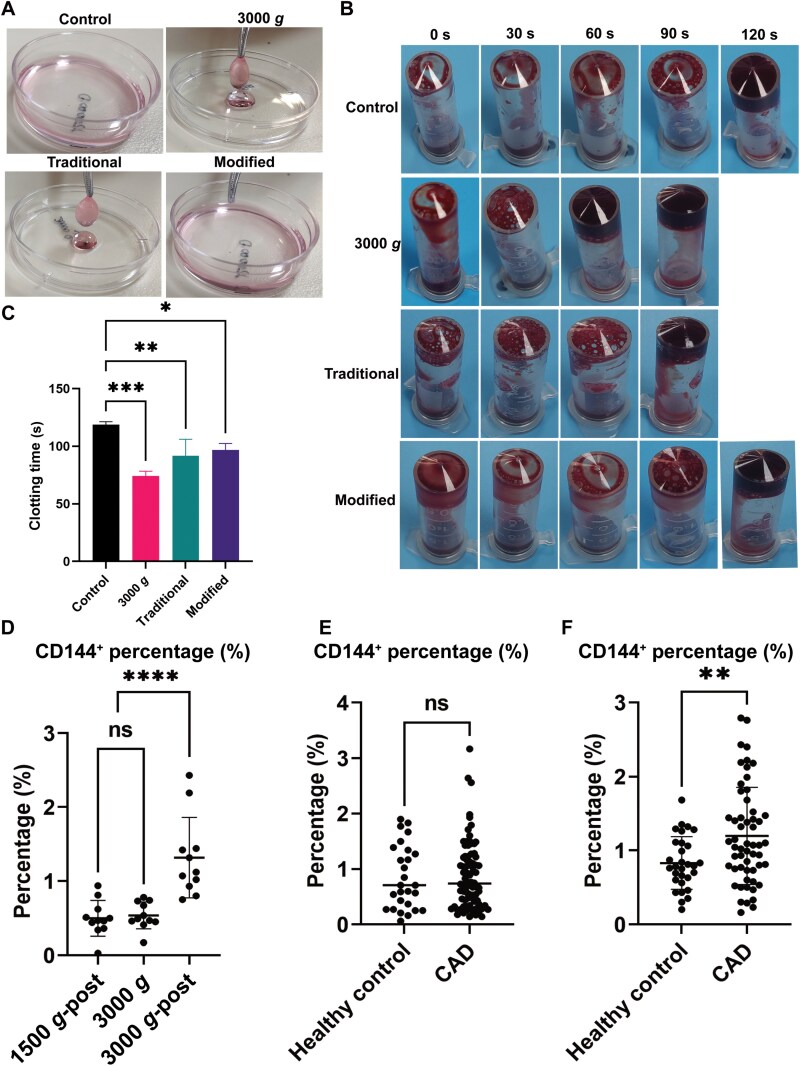
The roles of MVs in procoagulant activity and as biomarker of endothelial damage in CAD. (A) Normal cell cultured DMEM medium supplement with 10% FBS were treated with the indicated particles for 2 min, and then the clots were pick up with forceps. (B) *In vitro* coagulation test and clotting time (C) in different MVs groups. (D) CD144^+^ MVs were analyzed by flow cytometer for samples which obtained with 1500 *g*-post, 3000 *g* and 3000*g*-post protocols. (E, F) The difference of CD144^+^ MVs percentage between CAD and healthy individuals was shown for samples obtained with 1500 *g*-post protocols (E) and 3000 *g*-post (F) protocols. Data are mean ± SD. **P < 0.01, ****P < 0.0001.

Procoagulant activity and thrombus formation are well-known important function of MVs [8, 26]. To validate these functions, MVs isolated by different centrifugation protocols were added to general cell culture medium DMEM with 10% FBS. A clot was quickly formed in the medium that contained particles obtained using traditional centrifugation methods (1500 *g*-post) or 3000 *g*. However, the medium remained clear when MVs harvested with the modified protocol (3000 *g*-post) were added (Fig. 4A). Furthermore, the impact of MVs on procoagulant function and microthrombosis was evaluated via *in vitro* coagulation tests. As shown in Fig. 4B and 4C, obvious coagulation was observed at about 60–90 s in 3000 *g* and 1500 *g*-post treatments, but no significant differences were found between the control and 3000 *g*-post. This data suggests that the procoagulant function of 1500 *g*-post “MVs” described in many literatures is largely dependent on the presence of a large number of platelets and platelet debris, which can be isolated at 3000 *g*, rather than the true function of plasma membrane vesicle MVs that require higher speed centrifugation.

### Endothelial-derived MVs (EMVs) reflect coronary artery disease (CAD)

CAD is a major cause of mortality and morbidity across the world and is associated with a pro-inflammatory and pro-coagulant state of the body. Previous studies have demonstrated that the CD31^+^ EMP is a sensitive marker for CAD based on data from some groups [36, 37]. While the levels of CD144^+^ EMVs also increase in patients with CAD and predict CAD more strongly than traditional risk factors [38, 39]. The correlation between CD144^+^ EMVs and CAD was found to be poor and not significant [40]. In order to compare the traditional and modified methods of MVs isolation in diagnosing diseases, we first studied the CD144^+^ EMVs in patients with CAD. 93 patients with CAD who were scheduled for invasive angiography and percutaneous coronary intervention (PCI), as well as comparator group recruited from the physical examination center. As expected, the level of CD144^+^ EMVs harvested with the modified protocol was approximately five folds higher than with the traditional protocol and 3000 *g* (Fig. 4D). Interestingly, CAD patients had 30%–40% higher levels of CD144^+^ EMVs, compared to the healthy control in 3000 *g*-post group (Fig. 4F), while no significant difference was found in the 1500 *g*-post EMP (CD144^+^) group (Fig. 4E). These results suggest that the new microparticles may serve as a biomarker for enhanced vascular injury in individuals with CAD and provide a novel therapeutic target for CAD.

### Promote angiogenesis

**Figure 5.**
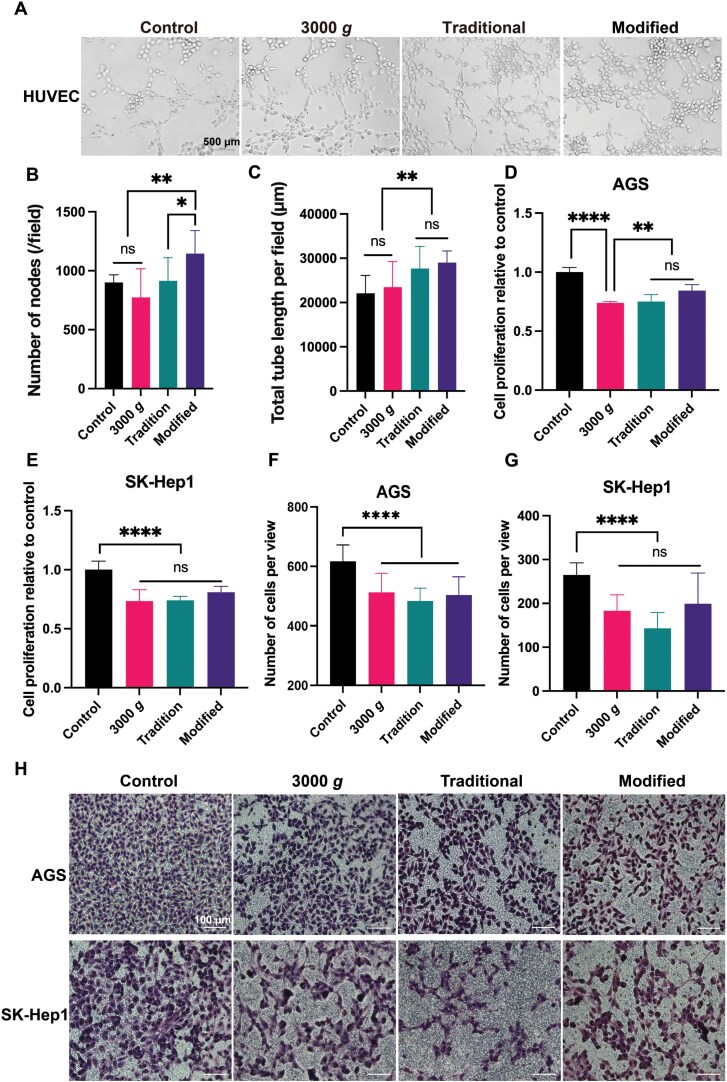
The effects of MVs on angiogenesis and cell migration. (A) Tube formation assay was performed in Matrigel and treated with indicated particles for 2 h, and representative images of the endothelial tubular networks were shown. (B) Quantification of the network characteristics like the number of tube nodes and total length of tubes (C) with Image J. (D, E) Cell proliferation was detected with MTT assay for AGS (D) and SK-Hep1 cells (E) after being treated with 3000 *g*, traditional and modified MVs for 48 h. (F–H) Transwell migration assay were treated with 3000 *g*, traditional and modified MVs for 24 h for AGS (F) and SK-Hep1 cells (G), which imaged with 5× magnification and quantified with Image J, and representative images were shown (H). Data are mean ± SD; *n* = 5 per group. *P < 0.1, **P < 0.01, ***P < 0.001.

MVs have been reported to exhibit proangiogenic activity by promoting the formation of capillary-like structures [9, 41]. To investigate the angiogenic effect of MVs, we performed a tube formation assay with human umbilical vein endothelial cells (HUVECs) cultured in Matrigel in the presence of MVs isolated by different protocols. The formation of tubular structures was imaged after being cultured on Matrigel for 2 h (Fig. 5A). Interestingly, compared to the control and 3000 *g* groups, the tubules of 3000 *g*-post group were denser, and some tubules were darker in both traditional and modified protocol groups, with the 3000 *g*-post showing more evident effects (Fig. 5A). Following imaging of the complexes, we analyzed the characteristic information of the networks to quantify and compare the angiogenesis potentiality among the groups. Consistent with previous reports, the groups treated with MVs isolated with modified and traditional methods had more evolved networks in terms of number of nodes and total length (Fig. 5B and 5C). Furthermore, the formation of tubes treated with MVs from the modified method was the best of all, while the characteristics of tubes treated with 3000 *g* particles were similar to those of non-treated control (Fig. 5B and 5C). These results suggested that the new MVs promote angiogenesis, but platelet-derived fragments are not or much weaker. Thus, it indicates that the previously reported the pro-angiogenic function is the function of modified MVs themself.

### Suppress tumor cell proliferation and migration, but promote endothelial cell migration

Some studies have reported that MVs could suppress cell proliferation and migration [35, 42]. We also performed an *in vitro* transmigration and MTT assay to investigate the effects of MVs on endothelial cell migration and proliferation. Interestingly, no differences were found in HUVEC cells proliferation after treatment with MVs obtained from different methods (Fig. S9A). Moreover, our results demonstrated that MVs obtained from both modified and traditional methods increased HUVEC cells migration, while the 3000 *g* sample did not show the similar effect (Fig. S9B and 9C). Thus, similar to promoting angiogenesis, the function of promoting endothelial cell migration may more likely belong to other cell-derived MVs, rather than platelet-derived particles.

Furthermore, MVs can interact with the extracellular matrix components to facilitating tumor cell proliferation and migration. Therefore, we investigated the effects of MVs on tumor cell proliferation, The data showed that MVs obtained from 3000 *g*, modified and traditional methods suppressed the proliferation of both AGS (gastric adenocarcinoma) and SK-Hep1 (hepatocellular carcinoma) cells (Fig. 5D and 5E). Furthermore, all of the MVs from different methods inhibited tumor cell migration based on transwell assay for AGS and SK-Hep1 (Fig. 5F–H). These findings suggested that all particles from these three methods suppress both tumor cell proliferation and migration. In summary, our findings show that MVs can suppress tumor cell proliferation and migration, while promoting endothelial cell migration. These differential effects suggest that the functional impact of MVs may depend on the specific cell types involved.

### Suppress the inflammatory responses

**Figure 6.**
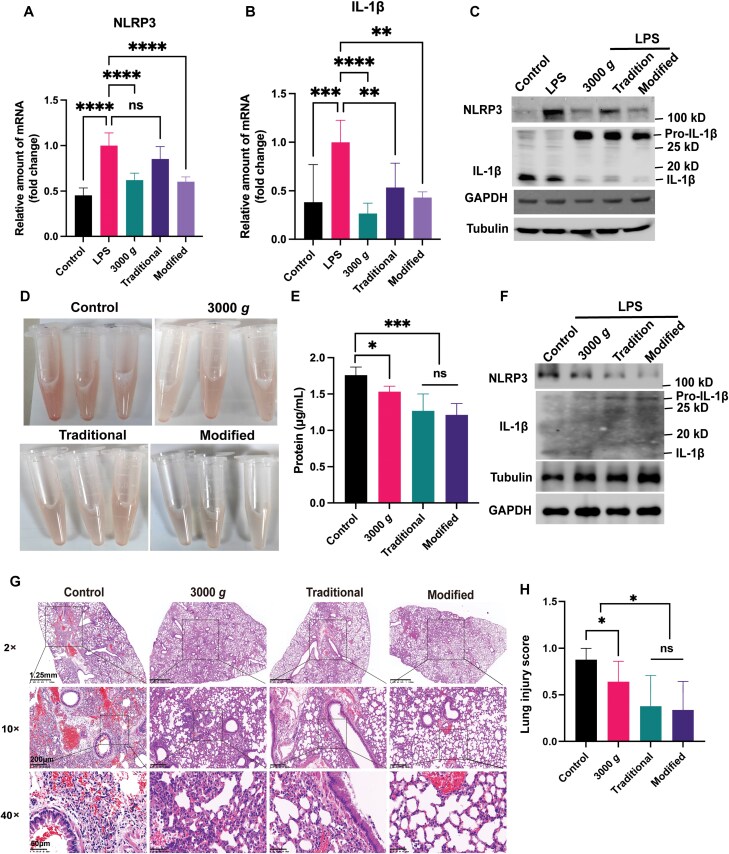
The effect of MVs on inflammatory response. (A–C) iBMDM cells were treated at indicated treatment for 6 h, and then harvested the samples and the mRNA level of NLRP3 (A) and IL-1β (B) were detected, and Western blotting to detect indicated proteins (C). (D) ALI mouse model obtained by being instilled i.t. LPS, isolated the MVs from the plasma of these mice 1 day later, and then instilled i.t. other mice together with LPS. 1 day later, BALF was harvested with 700 μL PBS. (E) Quantified the proteins in BALF from different treatments. (F) The cells from BALF were harvested and the levels of IL-1β and NLRP3 were detected with Western blotting. (G) The acute inflammatory regions in lung stained with H&E after being instilled i.t. LPS and treated with indicated MVs, and their quantification (H).

Although Garnier et al., reported that MVs isolated from sickle patient plasma could trigger a proinflammatory phenotype of endothelial cells [43]. Most of other studies found that the MVs suppress the inflammatory responses [23–25, 44]. In order to determine the effect of particles obtained by different methods on the inflammatory response, we first stimulated mice immortalized bone marrow-derived macrophage (iBMDM) with MVs *in vitro* and detected the expression of related inflammatory proteins. Interestingly, we found that after MVs treatment, the RNA levels of inflammatory factors IL-1βand its regulated protein NLRP3 were significantly decreased, much less than the positive control lipopolysaccharide (LPS) stimulation (Fig. 6A and 6B). The results showed that the protein level of IL-1β increased after MVs treatment, but only its precursor form increased, and its functional form of mature IL-1β was significantly lower than the control group and LPS treatment group, which may be due to the decrease of NLRP3 (Fig. 6C).

In addition, to further detect how MVs affected the inflammatory response, we used LPS to induce an acute lung injury (ALI) mouse model for inflammation. One day later, we instilled i.t. with particles obtained at 3000 *g*, 1500 *g*-post, and 3000 *g*-post to observe the effect of MVs on the inflammatory response. Compared with control mice, the color of bronchoalveolar lavage fluid (BALF) of the mice injected with MVs became significantly lighter, indicating that the hemoglobin content of them was greatly reduced, and the traditional and modified methods of higher speed centrifugation were especially more effective (Fig. 6D). The protein concentration in the BALF was detected, and found that the protein content of the groups injected with MVs or cell fragments was significantly lower than that of the control group (Fig. 6E). Furthermore, data showed that the protein level of IL-1β increased after MVs treatment, but only its precursor form increased, and its functional form of mature IL-1β was significantly lower than the control group, which may be due to the decrease of NLRP3 (Fig. 6F). Importantly, the inflammatory response in the injury lung in MVs treatment was improved compared to the control group. All mice instilled i.t. with MVs had fewer infiltrations of immune cells and improved endothelial permeability, especially the 3000 *g*-post obtained MVs treatment had a very significant anti-inflammation effect (Fig. 6G and 6H). Therefore, the results demonstrated that both MVs and platelet-derived particles can significantly suppress the inflammatory response in cells and mice. The modified MVs may have a much better effect because they contain much less proteins (about 20% of traditional MVs, as seen in Fig. 2G).

## Discussion

**Figure 7.**
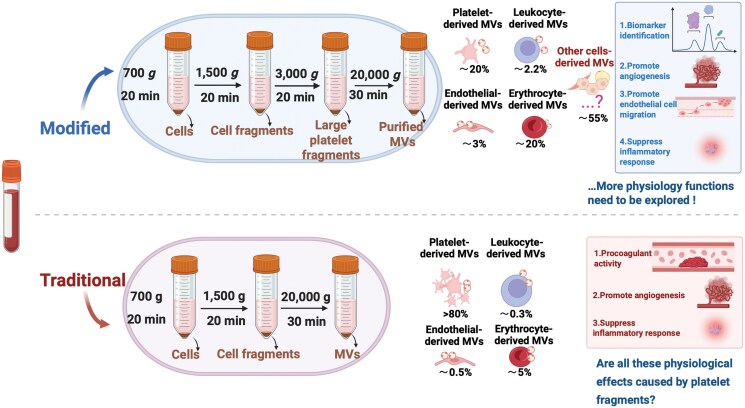
Graphical summary. Modified centrifugation enhances microvesicles purity and diversity, revealing broader physiological functions.

MVs were initially described as subcellular materials originating from platelets in normal plasma and serum [24]. The current diversity of isolated protocols for MVs results in a heterogeneous population of unknown origins. Moreover, the inclusion of additional structures into the pool of MVs, such as apoptotic bodies, migrasomes, or even exosomes, will further expand the pool of MVs [45]. In this study, we isolated MVs using different centrifugation speed intervals and performed a systematic analysis. We found that the isolation methods greatly affect MVs’ enumeration and functional properties, with 3000 *g* being a critical centrifugation speed in determining their composition (Fig. 7).

Our results showed that platelet-derived particles accounted for over 80% of the particle components obtained under 3000 *g*, while only about 20% of the MVs components obtained by centrifugation above 3000 *g* came from platelets (Figs. 1 and 2). The rest came from other cells of the body, indicating a significant increase in the proportion of MVs components derived from other cells other than platelets, as the proportion of other cell-derived MVs increases, their characteristic phenotype and function are enhanced, leading to more diverse functions. This shift in MVs’ composition also makes them more likely to become clinical diagnostic markers for certain diseases. In addition, the acquisition of plasma MVs is very simple and convenient, and in-depth research will undoubtedly have very important clinical significance, and it can also be an essential research direction for future research on MVs.

Furthermore, the markedly different components of MVs naturally lead to different functions. For example, contradicting previous literature that reported procoagulation as a primary function of MVs. We found that the procoagulant function of MVs greatly reduced or even disappeared after most of the small or large platelet fragments were removed by 3000 *g* centrifugation (Fig. 5A). On the other hand, MVs derived from other cells may have functions of promoting angiogenesis and cell migration, which were not observed in large platelet-derived particles. This finding clarifies previous misconceptions and provides new insights into the functions of MVs.

In addition, the simple and convenient acquisition of MVs has made them an attractive biomarker for the diagnosis and prognosis of many major diseases. However, traditional methods of isolation (1500 *g*-post) result in MVs derived from platelets accounting for 70%–90% of the isolated population, making it difficult to detect MVs from other cells. Therefore, this has hindered the identification of changes in MVs as diagnostic markers for the development of diseases in the past decades. Herein, our modified protocol decreased the proportion of platelet-derived MVs in the plasma to about 20%, while increasing the proportion of MVs from other cells by more than 5 folds. Our research showed that these new MVs can serve as excellent markers for the development and treatment of cardiovascular diseases. We are also exploring their diagnostic significance in other diseases, such as cancer and inflammation. Furthermore, our study found that MVs obtained by different centrifugation methods had the function of inhibiting inflammation, which may be mainly caused by platelet-derived particles. Our research showed that many functions of MVs may need to be re-validated, and it is important to clarify which cell-derived MVs mediated these functions to conduct in-depth research on their mechanisms. This may also be another important direction for MVs studies in the future.

## Research limitations

However, several limitations should be acknowledged. First, the clinical validation was conducted in a relatively small, single-center cohort of CAD patients and healthy controls; thus, larger, multicenter studies are warranted to confirm the translational value of the modified protocol. Second, while functional differences between MVs populations were observed, the precise molecular cargo and signaling mechanisms responsible for these effects remain to be elucidated.

## Methods

### Blood samples

Microparticles were prepared from healthy controls and CAD patients with informed consent under an approved protocol. Citrated blood (4 mL) was collected using a 21-gauge needle and processed within 2 h.

### The isolation of circulating MVs

Circulating MVs were obtained with three different centrifugation protocols as shown in Fig. 2A. Centrifugation for microvesicles isolation was performed using an Eppendorf 5417R benchtop centrifuge with a fixed-angle rotor. The centrifugation protocol 1 including two steps was performed according to the traditional protocol, which was centrifuged at 1500 *g* for 20 min and then 20,000 *g* for 30 min to precipitate MVs at 4°C. The protocol 2 includes: 1500 *g* was the same to remove cells and platelets, and then centrifuged at 3000 *g* to remove debris of cells, followed by 20,000 *g* for 30 min to get the MVs pellet; The protocol 3 followed as below: 1500, 3000, 5000, 8000, and 12000 *g* for 20 min, respectively, finally at 20,000 *g* for 20 min to pellet MVs. The pellets containing MVs were fixed or suspended in 10 μL phosphate buffer saline (PBS) in 1.5 mL centrifuge tube.

### Flow cytometry detection

The effect of centrifugation on the detection and quantification of MVs was analyzed by flow cytometry. The microparticle gate was set according to the size and were defined from 50 nm to 1000 nm in diameter. Flow cytometry was performed with a 12-color, triple laser BD FACS Lyric flow cytometry (BD Life Sciences, CA, 95131, USA) using BD FACSuite acquisition and analysis software to analyze data. MVs were isolated at different centrifugations and then fixed/permeabilized with BD Cytofix/Cytoperm (BD) for 30 min at 4°C, then washed three times with BD Perm/Wash (BD).

After blocking with Hu Fc Block (BD PMG), samples were incubated with antibodies against CD41a, CD45, CD235a, CD144, and COX4 for 30 min, then washed with BD Perm/Wash. The initial gating strategy for MVs is generally based on size by calibration beads. Beads of approximately 1 μm are used to set the upper size limit for MVs detection. Surface markers were normalized using isotype controls. All flow cytometric data were analyzed in Flowjo software.

### Clotting test in vitro

Particles isolated from human blood by different centrifugation protocols were added into the Dulbecco’s modified Eagle’s medium (DMEM) supplemented with 10% fetal bovine serum (FBS). Representative optical images were taken after 5 min.

As for *in vitro* coagulation test, harvested the 3000 *g*, 1500 *g*-post, and 3000 *g*-post particles from mouse blood as described as the above protocols, respectively. And then whole blood samples from C57BL/6 mouse were applied for *in vitro* coagulation test. Sample tubes containing 300 μL whole blood was loaded with 50 μL particles, which come from around 1 mL mouse plasma. The tubes were placed inverted to detect coagulation. And the clotting time was recorded.

### Immunofluorescent staining

MVs isolated in different centrifugations were fixed with 4% paraformaldehyde for 30 min, then washed three times in PBS. After blocking with 5% BSA, samples were stained with antibodies against CD41, CD45, and glycophorin at 4°C overnight. Secondary antibody staining was done in a blocking buffer at room temperature for 2 h. Samples were imaged using an LSM710 microscope (Zeiss) equipped with a polychromatic META detector.

### Electron microscopy of microparticle samples

TEM was used to visualize and measure sizes of MVs. In brief, 5 µL from each sample fraction was placed on a 200-mesh formvar and carbon-coated copper grid for 3 min. The excess solution was soaked off by a filter paper before the grid was stained with 5 μL of phosphotungstic acid for 1 min. Excess stain was wicked away to yield a dry grid which was prior to image acquisition by TEM (TALOS F200 × scanning/transmission electron microscope) operating at 120 kV.

### Dynamic light scattering (DLS)

MVs size was determined using the Zetasizer ZS (Malvern Panalytical, Malvern, UK) by being exposed to monochromatic light from a laser. MVs scatter the light and undergo Brownian motion which can be analyzed to provide the relative distribution from the different size in terms of how many particles are present. Briefly, samples of MVs were diluted in 1mL of PBS and were measured at 25°C. The analyzers presented microparticle size distribution graphically and in tabular form consisting of a mean size (nm), standard deviation (SD) and polydispersity index (PdI).

### Western blotting

The total proteins from MVs were extracted using RIPA lysis buffer containing phosphatase and protease inhibitors. BCA protein assay kit was used to determine the concentrations of extracted proteins. 10% SDS–PAGE gel was prepared for an equal amount of protein from each sample to run and then transferred to polyvinylidene difluoride (PVDF) membranes. PVDF membranes were blocked with 5% skim milk and incubated with primary antibodies (including CD41, CD36, Perilipin1, glycophorin, CD45, Tom20, NLRP3, IL-1β, cleaved Caspase9, iNOS, TNF-α, Tubulin, GAPDH and β-actin) at 4°C overnight, followed by incubation with secondary horseradish peroxidase-conjugated antibodies for 1 h at room temperature.

### Cell culture

Mice immortalized bone marrow-derived macrophage (iBMDM), HUVEC and Hela cells were cultured in DMEM supplemented with 10% FBS, 100 μg/mL streptomycin, and 100 U/mL penicillin, maintained at 37°C in a humidified atmosphere (5% CO_2_).

### Quantitative reverse transcription PCR

To assess the anti-inflammatory effect, iBMDM cells were seeded into 12-well plates, which were stimulated by LPS (100 μg/mL) and then incubated with MVs isolated in different centrifugation for 6 h. Total RNA of cell was extracted by Trizol reagent (Takara, Japan), and then transcribed into cDNA by Hifair® II 1st Strand cDNA Synthesis Kit (Yeasen, China). Next, qPCR was performed using Hieff qPCR SYBR Green Master Mix (Yeasen) on LightCycler® 480 Instrument II (Roche). The following qPCR conditions were used: 40 cycles of denaturation at 95°C for 10 s, annealing at 60°C for 30 s. A comparative threshold cycle method was used to analyze the Q-PCR data, where the amount of target was normalized to the endogenous reference of β-actin in each sample. The primer sequences used were as follows:

β-Actin: 5′-CGTTGACATCCGTAAAGACC, TAGGAGCCAGAGCAGTAATC-3′;
IL-1β: 5′-TTCAGGCAGGCAGTATCACTC, GAAGGTCCACGGGAAAGACAC-3′;
NLRP3: 5′-GCCGTCTACGTCTTCTTCCTTTC, CATCCGCAGCCAGTGAACAGAG-3′.

### Tube formation: extracellular matrix gel in vitro angiogenesis assays

HUVEC were used for tube formation assay. Matrigel (Corning, NY, USA) was melted at 4°C and coated into 6-well plates with 500 µL per well under sterile conditions without introducing air bubbles. Let the plates sit at 37°C for at least 15 min to allow gelling of the Matrigel. HUVEC (1 × 10^5^ cells/well) were then seeded into the pre-coated 6-well plate. Add the MVs in a total volume of 20 µL per well at the same time. The tube-like structures are observed under a microscope at 2 h after incubation, and pictures can be taken. Total tube length and individual branch point number were assessed with ImageJ software.

### Transwell assay

In this assay, about 5 × 10^4^ HUVEC cells in 300 μL FBS-free medium was seeded on top of the filter membrane in a transwell, then inserted into the outer compartment of each well, which contained 750 μL medium with MVs, and incubated for 16 h. After incubation, the cells on the top of the membrane were gently scraped off using a cotton swab or a pipette tip to remove any non-migrated cells. The remaining migrated cells on the lower side of the filter membrane were then fixed and stained with Hematoxylin and Eosin. Pictures were captured and assessed with ImageJ software.

### Anti-inflammatory effect of MVs in LPS-induced acute lung injury in vivo

C57BL/6 mice (male, eight weeks) were obtained from the Animal Center of Shanghai Jiao Tong University School of Medicine. The experimental protocol was approved by the Animal Care Committees of Shanghai Jiao Tong University School of Medicine. Mice were anesthetized with an intraperitoneal injection of ketamine (100 mg/kg), and 1 μg ‘ultrapure’ LPS (*E. coli* O111:B4) in 100 μL was instilled intratracheally. Citrated blood was collected from LPS-treated mice after 24 h. MVs were isolated using three different centrifugation protocols as described before. And then MVs were washed three times to remove the remaining stimulatory factors. Washed MVs were then instilled intratracheally into the new batch of LPS-induced acute lung injury mice. 24 h after instillation, mice were euthanized, and bronchoalveolar lavage fluid (BALF) samples were analyzed for total protein levels using Bradford assay.

### Research ethics

Mice: The experiment was carried out with the approval of the Institutional Animal Care & Use Committee (IACUC) of Shanghai Jiao Tong University School of Medicine (Approval No. B-2021-009).

### Patients

This study involving human participants was reviewed and approved by the Ethics Committee of Ruijin Hospital (Approval No. 2018-183). Written informed consent was obtained from all participants in accordance with the Declaration of Helsinki.

### Statistical analysis

Statistical analyses were conducted using Sigma Plot version 14.0 and GraphPad Prism 9.0 (GraphPad, La Jolla, CA, USA). Quantitative variables are expressed as mean ± SD or median, whereas categorical variables are expressed as numbers and percentages. The Mann–Whitney *U*-test and ANOVA were used to compare the differences in two or more groups when appropriate. *P* < 0.05 was considered statistically significant.

## Supplementary Material

lnaf017_suppl_Supplementary_Materials

## Data Availability

All data needed to evaluate the conclusions in the paper are present in the paper and/or the Supplementary Materials.
