# Supplementary material for: The new composition of circulating microvesicles: optimized protocols and reassessment of their characteristics and physiological functions
Source: Life Med. 2025 Jun 11;4(4):lnaf017. doi: 10.1093/lifemedi/lnaf017 (PMC12277571; doi:10.1093/lifemedi/lnaf017)
Supplement: lnaf017_suppl_Supplementary_Materials [file lnaf017_suppl_supplementary_materials.docx]

**The new composition of circulating microvesicles: optimized protocols and reassessment of their characteristics and physiological functions**

Chen Zhang^1,2,3,5,#^ , Jiajia Hu^4,#^, Yifan Shi^1,2,3, #^, Yang Feng^1,2,3^, Zeyang Li^1,2,3^, Zi Dong^1,2,3^, Yiding Tang^1,2,3^, Guang Ning^1,2,*^, Zhengting Wang^5,*^, Guorui Huang^1,2,3,*^

^1^Department of Endocrine and Metabolic Diseases, Shanghai Institute of Endocrine and Metabolic Diseases, Ruijin Hospital, Shanghai Jiao tong University School of Medicine, Shanghai 200025, China

^2^Shanghai National Clinical Research Center for Metabolic Diseases, Key Laboratory for Endocrine and Metabolic Diseases of the National Health Commission of the PR China, Shanghai Key Laboratory for Endocrine Tumor，Ruijin Hospital, Shanghai Jiao tong University School of Medicine, Shanghai 200025, China

^3^National Research Center for Translational Medicine, State Key Laboratory of Medical Genomics, Ruijin Hospital, Shanghai Jiao tong University School of Medicine, Shanghai 200025, China

^4^Department of Nuclear Medicine, Ruijin Hospital, Shanghai Jiao tong University School of Medicine, Shanghai 200025, China

^5^Department of Gastroenterology, Ruijin Hospital, Shanghai Jiao tong University School of Medicine, Shanghai 200025, China

^#^These authors contributed equally to this work.

^*^Correspondence: hgr12038@rjh.com.cn (G.H.), zhengtingwang@shsmu.edu.cn (Z.W.), gning@sibs.ac.cn (G.N.),

**Table 1. Characteristics of MPs with different published centrifugation protocols.**

| **Step 1** | **Step 2** | **MPs’ function** | **Sample** | **Ref.** |
| --- | --- | --- | --- | --- |
| 200 ×*g*, 10 min | 10,000 ×*g*,10 min | Promote inflammatory signaling in acute lung injury | Murine BALF | ^1^ |
| 1000 ×*g*, 10 min | 14,000 ×*g*,60 min | Promote lung metastasis^2^; mediate tumor eradication^3^ | Tumor cells | ^2,3^ |
| 1000 ×*g*, 10min | 20,817 ×*g*, 20 min | Trigger a proinflammatory phenotype of ECs | Blood | ^4^ |
| 1100 ×*g*, 15 min | 7,000 ×*g*, 3 min | Biomarker for cancer | Blood | ^5^ |
| 1500 ×*g*, 15min | 12,000 ×*g*, 50 min | Trigger proinflammatory response and apoptosis | Lymph blastoma cells | ^6^ |
| 1500 ×*g*, 15min | 13,000 ×*g*, 2 min | Procoagulant activity ^7-9^; improve angiogenesis ^10^; biomarker for ARDS ^11^ | Blood ^7-10^; BALF [16] | ^7-11^ |
| 1500 ×*g*, 5 min | 15,000 ×*g*, 30 min | Response to decompression. | Blood | ^12^ |
| 1500 ×*g*, 20 min | 18,000 ×*g*, 30 min | Thrombus ^13,14^ and ROS formation ^15^; biomarkers for COPD^16^ and asthma ^17^ | Blood | ^13-18^ |
| 1500 ×*g*, 10 min | 100,000 ×*g*, 30 min | Proteolytic activity ^19^; procoagulant activity ^20^ | THP-1 monocytes | ^19,20^ |
| 1800 ×*g*, 10 min | 100,000 ×*g*, 120 min | Propagate coagulation | BALF | ^21^ |
| 2000 ×*g*, 20 min | 16,000 ×*g*, 40 min | Suppress the inflammatory responses | Murine BALF | ^22^ |
| 2000 ×*g*, 6 min and 3000 ×*g*, 2 min | 20,800 ×*g*, 60 min | Modify endothelial cell functions ^23^; promote microvascular sludging ^24^ | Blood | ^23,24^ |
| 2000 ×*g*, 15 min | 100,000 ×*g*, 30 min | Propagate coagulation | Blood | ^25^ |
| 2000 ×*g*, 20min | 200,000 ×*g*, 60 min | Pathogenesis of atherosclerosis and diabetes [31-32]; immune response [33] | Blood | ^26-28^ |
| 2500 ×*g*, 15 min | 19,800 ×*g*, 10 min | Improve angiogenesis, blood flow and ischemic recovery | Blood | ^29^ |
| 2600 ×*g*, 20min | 19,800 ×*g*, 40 min | Trigger proinflammatory response | Rat blood | ^30^ |
| 3200 ×*g*, 10 min | 20,000 ×*g*, 90 min | Carriers of functional Ago2 microRNA complexes | Blood | ^31^ |
| 5000 ×*g*, 15 min | 20,000 ×*g*, 30 min | Glomerular endothelium dysfunction ^32^; procoagulant activity ^33^ | Rat blood | ^32,33^ |
| 5000 ×*g*, 10 min | 100,000 ×*g*, 20 min | Suppress cell migration and  Proliferation ^34^; trigger proinflammatory response ^35^ | Platelet ^34^; endothelial cells^35^ | ^34,35^ |

1 Mohning, M. P. *et al.* Phagocytosis of microparticles by alveolar macrophages during acute lung injury requires MerTK. *Am J Physiol Lung Cell Mol Physiol* **314**, L69-L82, doi:10.1152/ajplung.00058.2017 (2018).

2 Zhang, H. *et al.* Circulating Tumor Microparticles Promote Lung Metastasis by Reprogramming Inflammatory and Mechanical Niches via a Macrophage-Dependent Pathway. *Cancer Immunol Res* **6**, 1046-1056, doi:10.1158/2326-6066.CIR-17-0574 (2018).

3 Wan, C. *et al.* Irradiated tumor cell-derived microparticles mediate tumor eradication via cell killing and immune reprogramming. *Sci Adv* **6**, eaay9789, doi:10.1126/sciadv.aay9789 (2020).

4 Curtis, A. M. *et al.* p38 mitogen-activated protein kinase targets the production of proinflammatory endothelial microparticles. *J Thromb Haemost* **7**, 701-709, doi:10.1111/j.1538-7836.2009.03304.x (2009).

5 Mege, D. *et al.* The origin and concentration of circulating microparticles differ according to cancer type and evolution: A prospective single-center study. *Int J Cancer* **138**, 939-948, doi:10.1002/ijc.29837 (2016).

6 Qiu, Q., Xiong, W., Yang, C., Gagnon, C. & Hardy, P. Lymphocyte-derived microparticles induce bronchial epithelial cells' pro-inflammatory cytokine production and apoptosis. *Mol Immunol* **55**, 220-230, doi:10.1016/j.molimm.2013.01.017 (2013).

7 Bal, L. *et al.* Factors influencing the level of circulating procoagulant microparticles in acute pulmonary embolism. *Arch Cardiovasc Dis* **103**, 394-403, doi:10.1016/j.acvd.2010.06.005 (2010).

8 Dignat-George, F. *et al.* Endothelial microparticles: a potential contribution to the thrombotic complications of the antiphospholipid syndrome. *Thromb Haemost* **91**, 667-673, doi:10.1160/TH03-07-0487 (2004).

9 Steppich, B. *et al.* Tissue factor pathway inhibitor on circulating microparticles in acute myocardial infarction. *Thromb Haemost* **93**, 35-39, doi:10.1160/TH04-06-0393 (2005).

10 Sheu, J. J. *et al.* Administered circulating microparticles derived from lung cancer patients markedly improved angiogenesis, blood flow and ischemic recovery in rat critical limb ischemia. *J Transl Med* **13**, 59, doi:10.1186/s12967-015-0381-8 (2015).

11 Guervilly, C. *et al.* High levels of circulating leukocyte microparticles are associated with better outcome in acute respiratory distress syndrome. *Crit Care* **15**, R31, doi:10.1186/cc9978 (2011).

12 Garnier, Y. *et al.* Plasma microparticles of sickle patients during crisis or taking hydroxyurea modify endothelium inflammatory properties. *Blood* **136**, 247-256, doi:10.1182/blood.2020004853 (2020).

13 Biro, E. *et al.* Human cell-derived microparticles promote thrombus formation in vivo in a tissue factor-dependent manner. *J Thromb Haemost* **1**, 2561-2568, doi:10.1046/j.1538-7836.2003.00456.x (2003).

14 Aharon, A., Katzenell, S., Tamari, T. & Brenner, B. Microparticles bearing tissue factor and tissue factor pathway inhibitor in gestational vascular complications. *J Thromb Haemost* **7**, 1047-1050, doi:10.1111/j.1538-7836.2009.03342.x (2009).

15 Burger, D. *et al.* Endothelial microparticle formation by angiotensin II is mediated via Ang II receptor type I/NADPH oxidase/ Rho kinase pathways targeted to lipid rafts. *Arterioscler Thromb Vasc Biol* **31**, 1898-1907, doi:10.1161/ATVBAHA.110.222703 (2011).

16 Takahashi, T. *et al.* Annual FEV1 changes and numbers of circulating endothelial microparticles in patients with COPD: a prospective study. *BMJ Open* **4**, e004571, doi:10.1136/bmjopen-2013-004571 (2014).

17 Duarte, D. *et al.* Increased circulating platelet microparticles as a potential biomarker in asthma. *Allergy* **68**, 1073-1075, doi:10.1111/all.12190 (2013).

18 Jy, W. *et al.* Measuring circulating cell-derived microparticles. *J Thromb Haemost* **2**, 1842-1851, doi:10.1111/j.1538-7836.2004.00936.x (2004).

19 Li, C. J. *et al.* Novel proteolytic microvesicles released from human macrophages after exposure to tobacco smoke. *Am J Pathol* **182**, 1552-1562, doi:10.1016/j.ajpath.2013.01.035 (2013).

20 Bastarache, J. A., Fremont, R. D., Kropski, J. A., Bossert, F. R. & Ware, L. B. Procoagulant alveolar microparticles in the lungs of patients with acute respiratory distress syndrome. *Am J Physiol Lung Cell Mol Physiol* **297**, L1035-1041, doi:10.1152/ajplung.00214.2009 (2009).

21 Novelli, F. *et al.* Procoagulant, tissue factor-bearing microparticles in bronchoalveolar lavage of interstitial lung disease patients: an observational study. *PLoS One* **9**, e95013, doi:10.1371/journal.pone.0095013 (2014).

22 Zhang, D. *et al.* A potential role of microvesicle-containing miR-223/142 in lung inflammation. *Thorax* **74**, 865-874, doi:10.1136/thoraxjnl-2018-212994 (2019).

23 Faille, D. *et al.* Endocytosis and intracellular processing of platelet microparticles by brain endothelial cells. *J Cell Mol Med* **16**, 1731-1738, doi:10.1111/j.1582-4934.2011.01434.x (2012).

24 Faille, D. *et al.* Platelet microparticles: a new player in malaria parasite cytoadherence to human brain endothelium. *FASEB J* **23**, 3449-3458, doi:10.1096/fj.09-135822 (2009).

25 Van Der Meijden, P. E. *et al.* Platelet- and erythrocyte-derived microparticles trigger thrombin generation via factor XIIa. *J Thromb Haemost* **10**, 1355-1362, doi:10.1111/j.1538-7836.2012.04758.x (2012).

26 Zhang, X. *et al.* Platelet-derived microparticle count and surface molecule expression differ between subjects with and without type 2 diabetes, independently of obesity status. *J Thromb Thrombolysis* **37**, 455-463, doi:10.1007/s11239-013-1000-2 (2014).

27 Zhang, X. *et al.* Oat-enriched diet reduces inflammatory status assessed by circulating cell-derived microparticle concentrations in type 2 diabetes. *Mol Nutr Food Res* **58**, 1322-1332, doi:10.1002/mnfr.201300820 (2014).

28 Dinkla, S. *et al.* Platelet microparticles inhibit IL-17 production by regulatory T cells through P-selectin. *Blood* **127**, 1976-1986, doi:10.1182/blood-2015-04-640300 (2016).

29 Yang, S. *et al.* Angiotensin II receptor type 1 autoantibodies promote endothelial microparticles formation through activating p38 MAPK pathway. *J Hypertens* **32**, 762-770, doi:10.1097/HJH.0000000000000083 (2014).

30 Lee, S. K., Yang, S. H., Kwon, I., Lee, O. H. & Heo, J. H. Role of tumour necrosis factor receptor-1 and nuclear factor-kappaB in production of TNF-alpha-induced pro-inflammatory microparticles in endothelial cells. *Thromb Haemost* **112**, 580-588, doi:10.1160/TH13-11-0975 (2014).

31 Laffont, B. *et al.* Activated platelets can deliver mRNA regulatory Ago2*microRNA complexes to endothelial cells via microparticles. *Blood* **122**, 253-261, doi:10.1182/blood-2013-03-492801 (2013).

32 Zhang, Y. *et al.* Platelet Microparticles Mediate Glomerular Endothelial Injury in Early Diabetic Nephropathy. *J Am Soc Nephrol* **29**, 2671-2695, doi:10.1681/ASN.2018040368 (2018).

33 Dasgupta, S. K., Le, A., Chavakis, T., Rumbaut, R. E. & Thiagarajan, P. Developmental endothelial locus-1 (Del-1) mediates clearance of platelet microparticles by the endothelium. *Circulation* **125**, 1664-1672, doi:10.1161/CIRCULATIONAHA.111.068833 (2012).

34 Schubert, P. *et al.* Releasates of riboflavin/UV-treated platelets: Microvesicles suppress cytokine-mediated endothelial cell migration/proliferation. *Transfusion* **61**, 1551-1561, doi:10.1111/trf.16337 (2021).

35 Brett, K. D. *et al.* Microparticle and interleukin-1beta production with human simulated compressed air diving. *Sci Rep* **9**, 13320, doi:10.1038/s41598-019-49924-1 (2019).


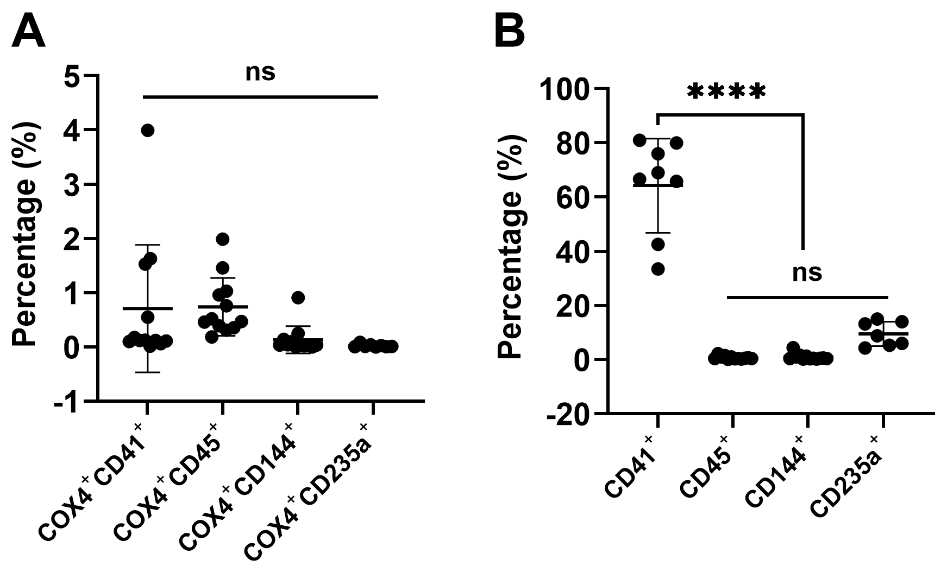


**Figure S1.** **Detection of circulating MVs with mitochondrial positive (Cox4^+^) (A) or cell-derived (B).**

**
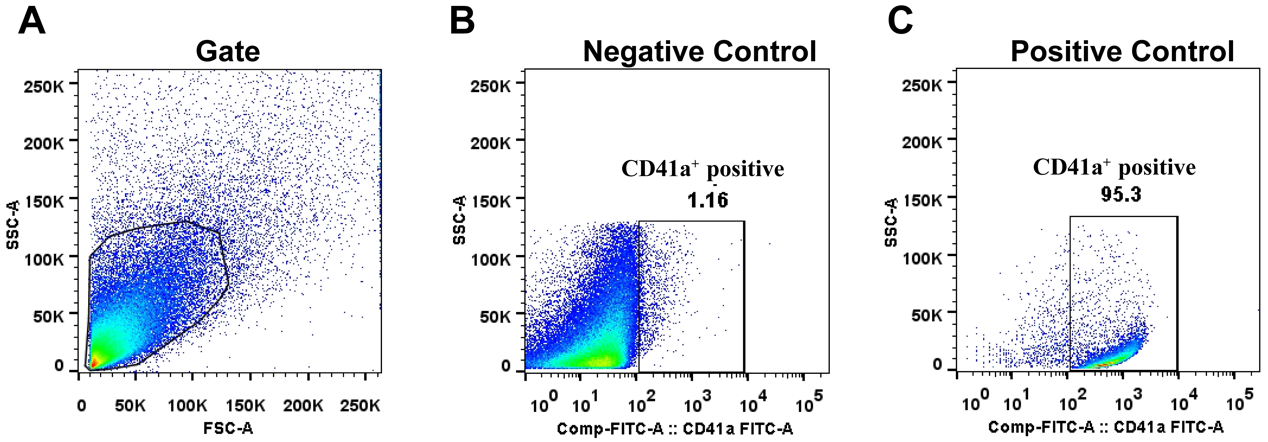
**

**Figure S2. The gate (A), CD41a^+^ negative (B) and positive (C) controls for flow cytometry analysis.**


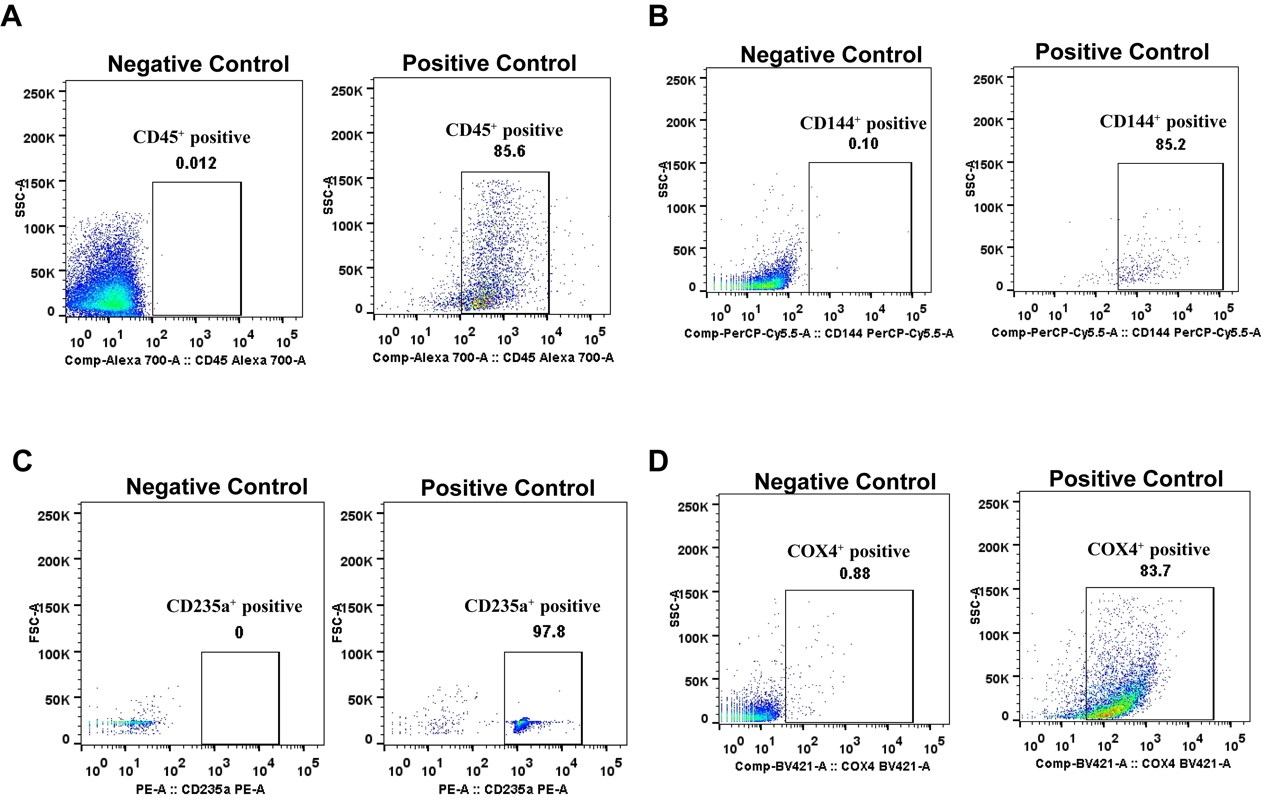


**Figure S3. The** **negative and positive controls CD45^+^ (A), CD144^+^ (B), CD235a^+^ (C) and Cox4^+^ (D) for flow cytometry analysis.**

**
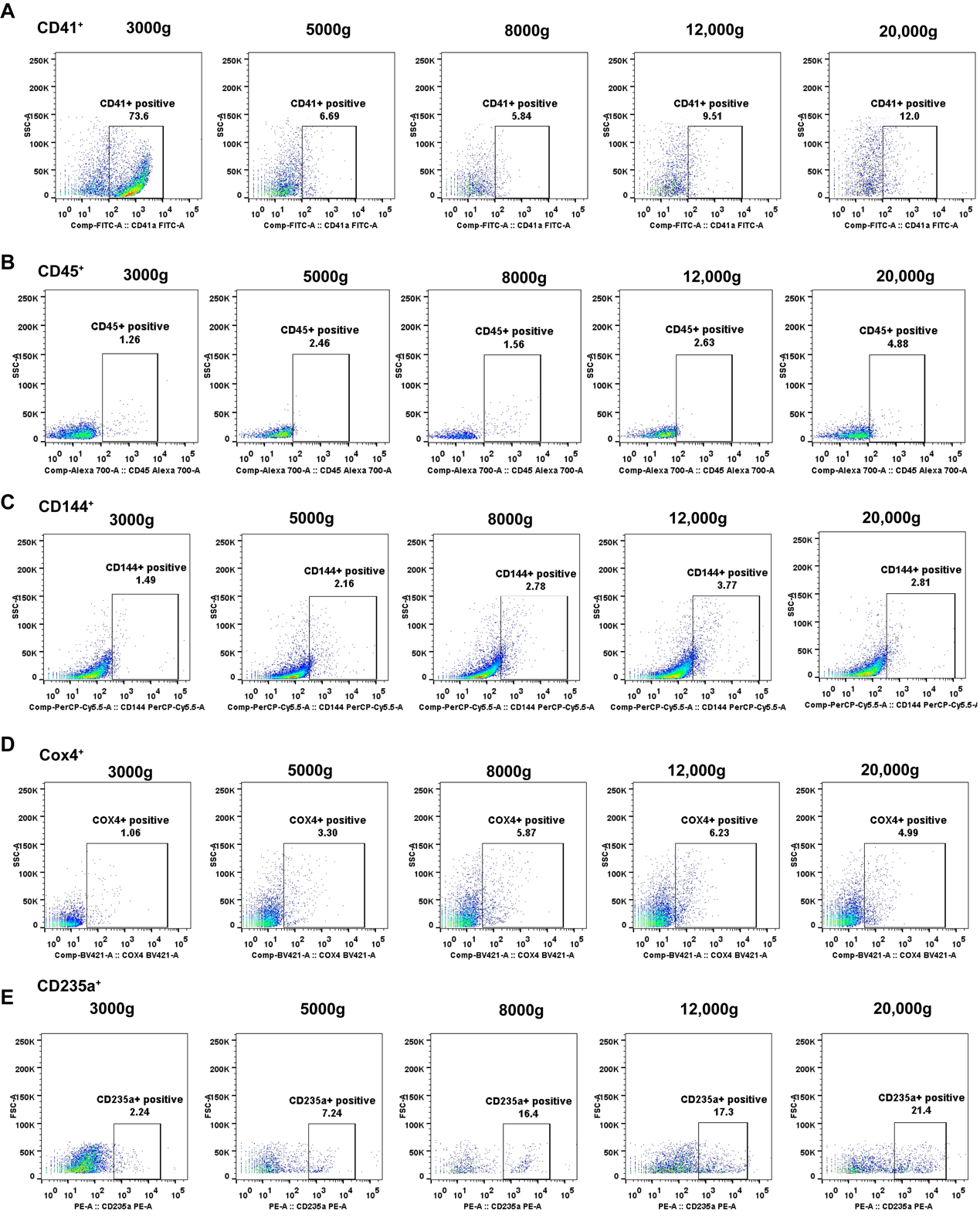
**

**Figure S4. Detection of circulating cell-derived MVs at centrifugal speed intervals.**

(A–E) After being centrifugated under different speeds intervals, the representative images of flow cytometry analysis for platelet-derived (CD41a^+^) (A), leukocyte-derived (CD45^+^) (B), vascular endothelial cells-derived (CD144^+^) (C), mitochondrial-derived (COX4^+^) (D) and erythrocyte-derived (CD235a^+^) MVs (E), were shown, respectively. *n* = 8–10 per group, data are presented as mean ± SD. ****P* < 0.001, *****P* < 0.0001.


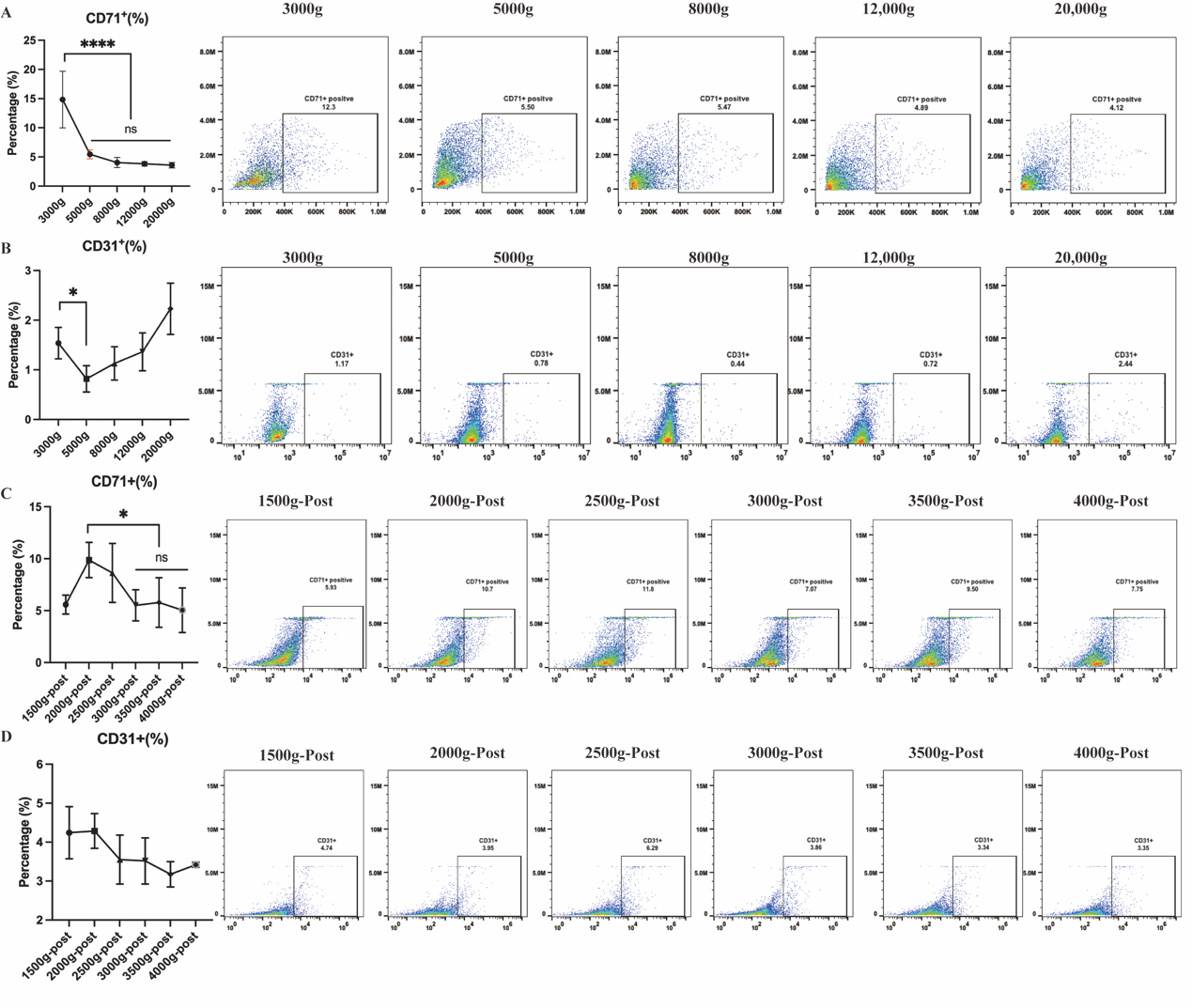


**Figure S5. Detection of circulating cell-derived MVs using another marker at centrifugal speed intervals.**

After being centrifugated under different speeds intervals, the run charts and representative images of flow cytometry analysis for platelet-derived (CD71^+^) (A), vascular endothelial cells-derived (CD31^+^) (B) at centrifugal speed intervals. Representative images of flow cytometry analysis for CD71^+^ MVs (C) and CD31^+^ MVs (D) were isolated at 20,000 *g* after initial concentration speeds of 1500 *g*, 2000 *g*, 2500 *g*, 3000 *g*, 3500 *g* and 4000 *g*-post, respectively.

*n* = 8–10 per group, data are presented as mean ± SD. ****P* < 0.001, *****P* < 0.0001.


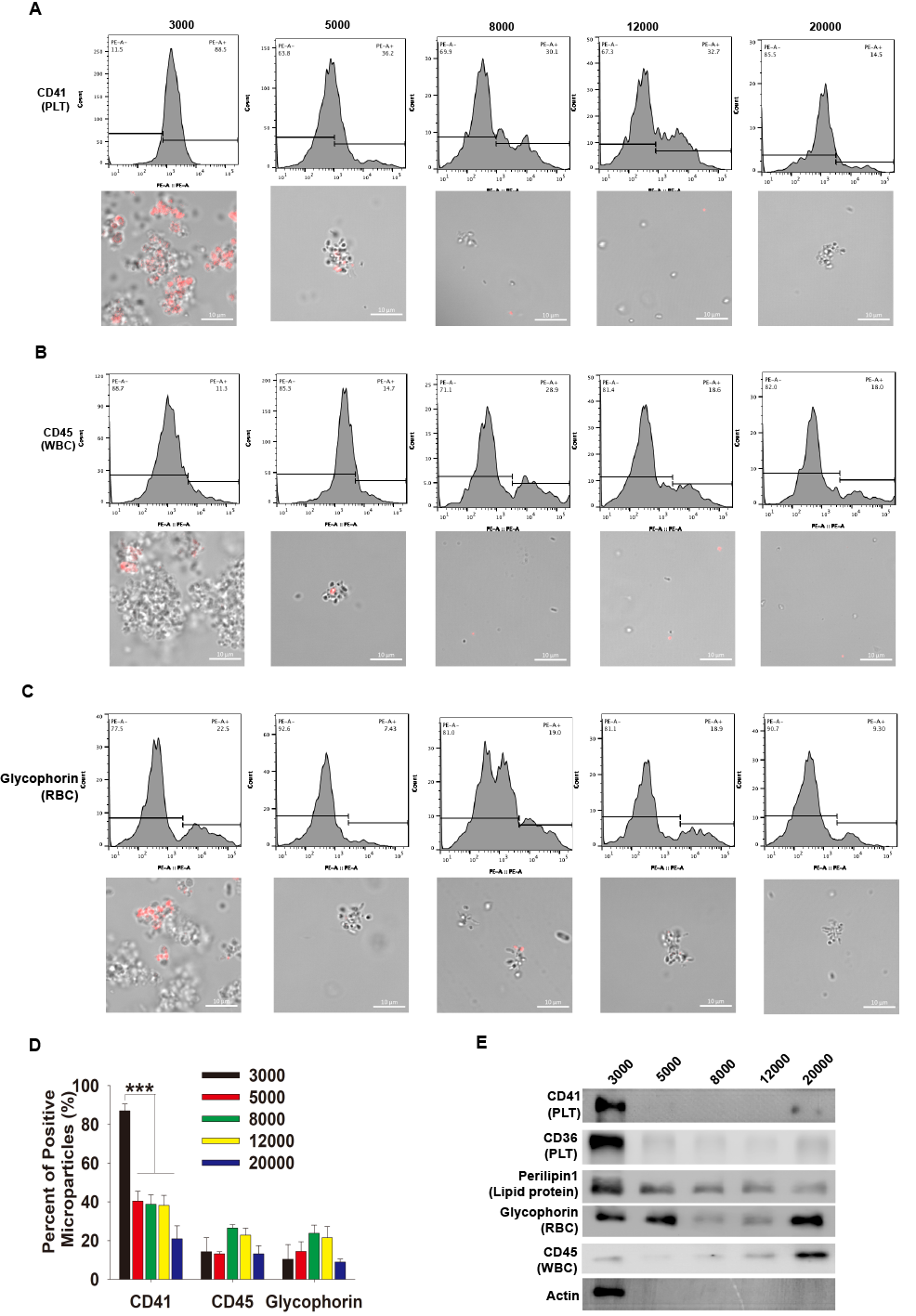


**Figure S6. Validation of circulating MVs origins at different centrifugal intervals.**

(A–C) Representative images of immunofluorescent staining and flow cytometry analysis for platelet-derived (CD41^+^) (A), leukocyte-derived (CD45^+^) (B) and erythrocyte-derived particles (glycophorin) (C) at indicated five different centrifugation speeds. (D) Statistical analysis of CD41, CD45 and glycophorin positive MVs at indicated centrifugal speeds. (E) Western blotting analysis of particles were isolated under different centrifugation speeds with antibodies against CD41, CD36, Perilipin1, glycophorin, CD45 and Actin. Data are mean ± SD; *n* = 4 per group. ****P* < 0.001.


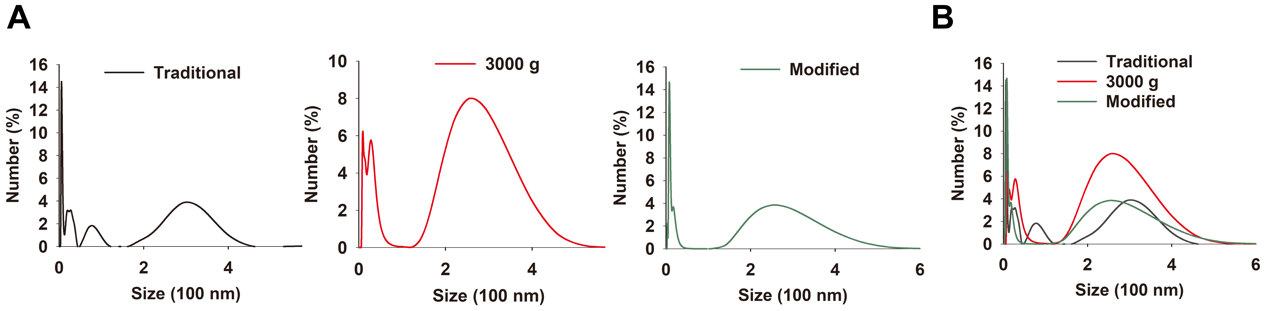


**Figure S7. The size distribution of MVs was analyzed by the dynamic light scattering (DLS) for indicated samples after being prepared with TEM methods.**


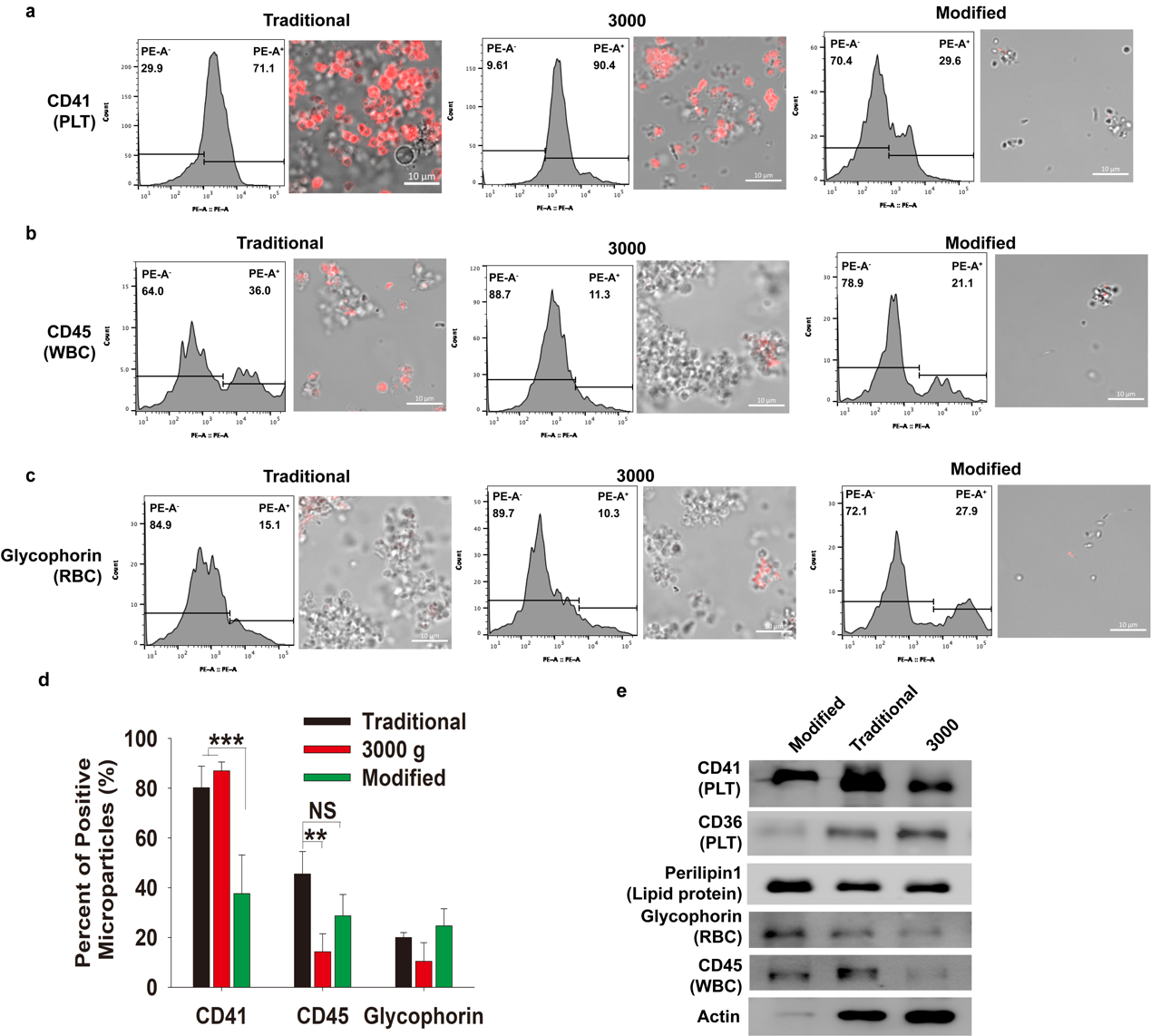


**Figure S8. Composition of isolated MVs with different centrifugations.**

(A–C) Immunofluorescent staining and flow cytometer analyze for the MVs from different cell-derived markers like CD41 (A), CD45 (B) and glycophorin (C) under traditional, 3000 *g* and modified centrifugation protocols. (D) Statistical analysis of the percentage of CD41, CD45 and glycophorin positive MVs at different speeds. (E) Western blotting analysis for particles isolated under indicated speeds with antibodies against CD36, CD45, glycophorin, Perillipin1, and cytoskeleton protein Actin. Data are mean ± SD; *n* = 4 per group. ***P* < 0.01, ****P* < 0.001.


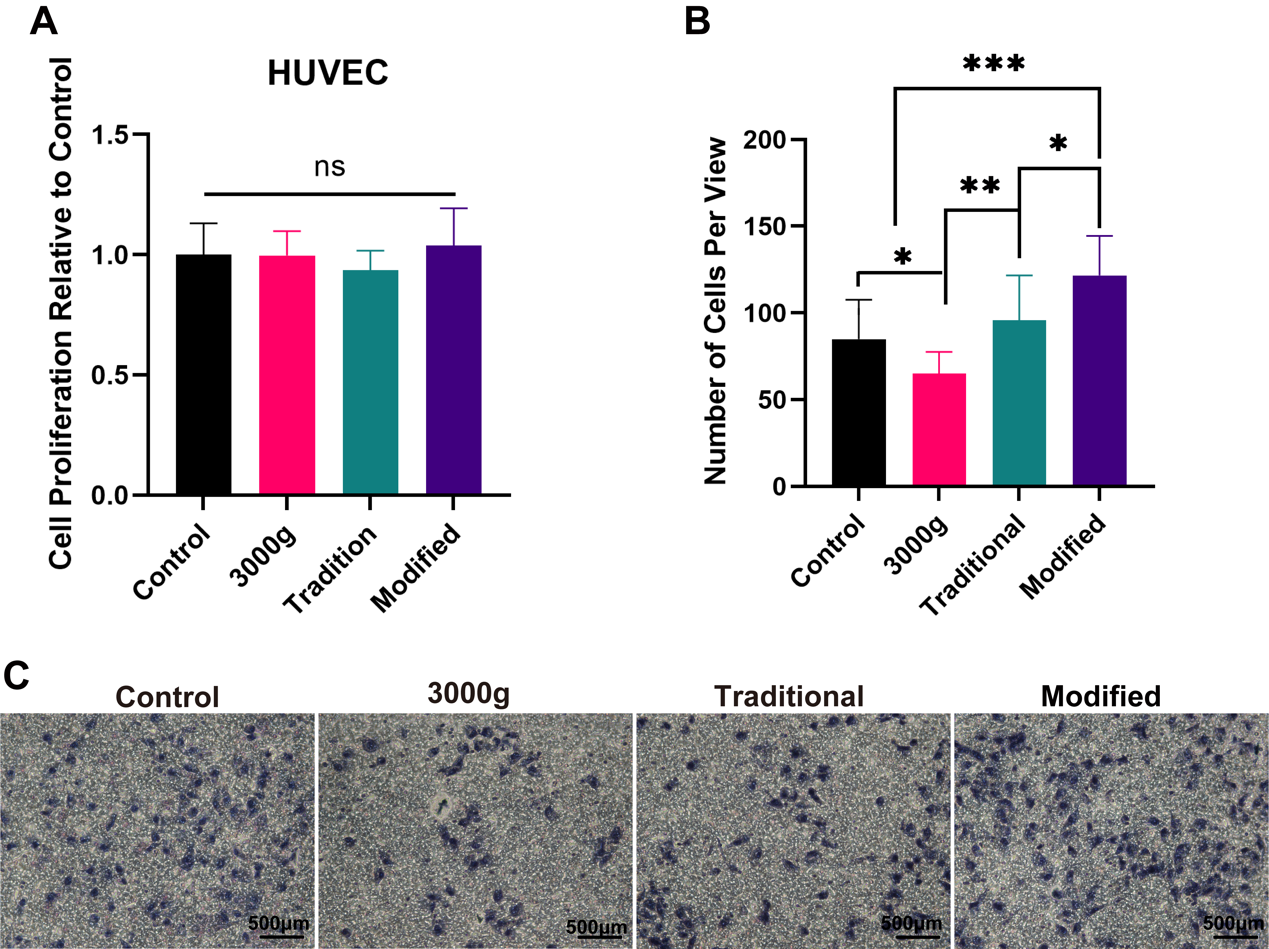


**Figure S9. The effects of different MVs on HUVEC cell proliferation and migration.**

(A) Cell proliferation was detected with MTT assay for HUVEC cells after being treated with 3000g, traditional and modified MVs for 48 h. (B, C) For transwell migration assay HUVEC cells were treated with 3000 *g*, traditional and modified MVs for 24 h, which imaged with 5× magnification and quantified with Image J (B), and representative images were shown (C). Data are mean ± SD; *n* = 5 per group. **P* < 0.05, ***P* < 0.01, ***P* < 0.001.
